# Supplementary material for: Sequence Learning Induces Selectivity to Multiple Task Parameters in Mouse Somatosensory Cortex
Source: Curr Biol. 2021 Feb 8;31(3):473–485.e5. doi: 10.1016/j.cub.2020.10.059 (PMC7883307; doi:10.1016/j.cub.2020.10.059)
Supplement: Document S1. Figures S1–S3 [file mmc1.pdf]

Current Biology, Volume *31*

Supplemental Information

Sequence Learning Induces Selectivity to Multiple Task Parameters in Mouse Somatosensory Cortex

Michael R. Bale, Malamati Bitzidou, Elena Giusto, Paul Kinghorn, and Miguel Maravall

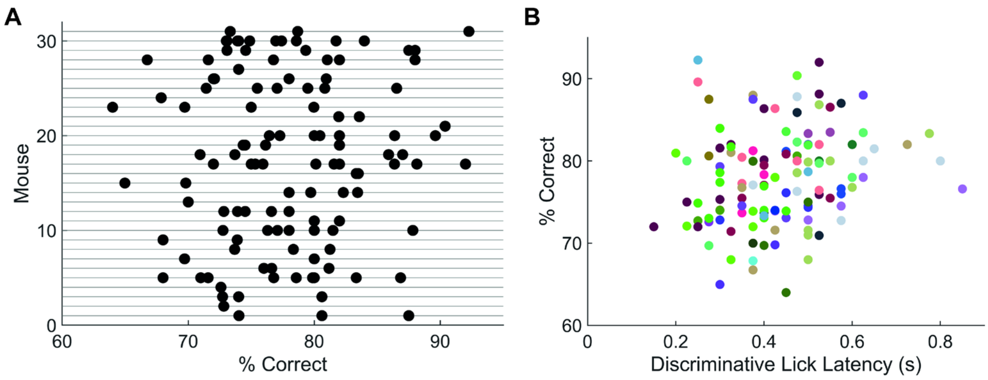

Figure S1. Discrimination of elementary tactile sequences in mice; related to Figure 1. (**A)** Performance (% correct) for all mice and sessions. Performance varied within and across mice. **(B)** Performance plotted against discriminative lick latency across all sessions, as in Figure 1F but with sessions for each mouse displayed in a different colour (n = 31 mice). Variability in performance and latency was present throughout the population of mice rather than being driven by specific animals.

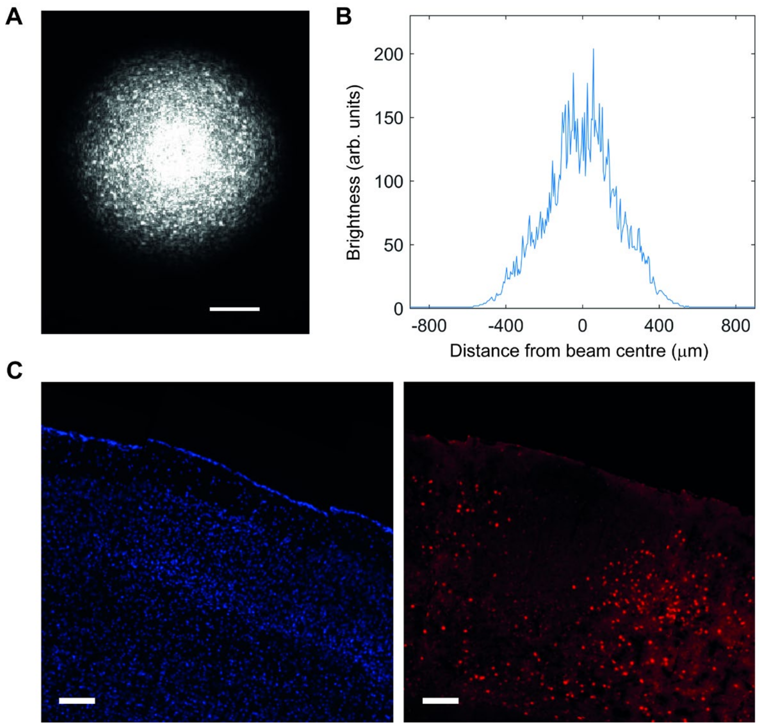

Figure S2. Tracking the participation of different cortical regions with optogenetic activity suppression; related to Figure 2. (A) Focused laser beam spot. Scale bar, 200 μm. **(B)** Measurement of laser beam profile (raw brightness reading, CMOS camera). **(C)** Immunohistochemical characterization of optogenetic suppression in VGAT-ChR2-EYFP mice. Fluorescence in the DAPI (left) and cFos (right) channel for an immunohistochemically processed S1bf slice, after optogenetic stimulation; scale bars, 100 μm. A region with weakened cFos but not DAPI expression can be clearly seen in the middle of the image and measured approximately 500 μm on the side (in the most superficial cortical layers) and 600 μm depth. We took the ratio of fluorescence intensity in the cFos channel and DAPI channel (whose spatially integrated intensity scaled with the density of neurons in an area), and compensated for potential non-specific changes in expression by normalising this ratio in the illuminated hemisphere (shown) to that in the corresponding contralateral region. Within the photoinhibited region, the cFos/DAPI ratio averaged 46% of its value in the contralateral hemisphere.

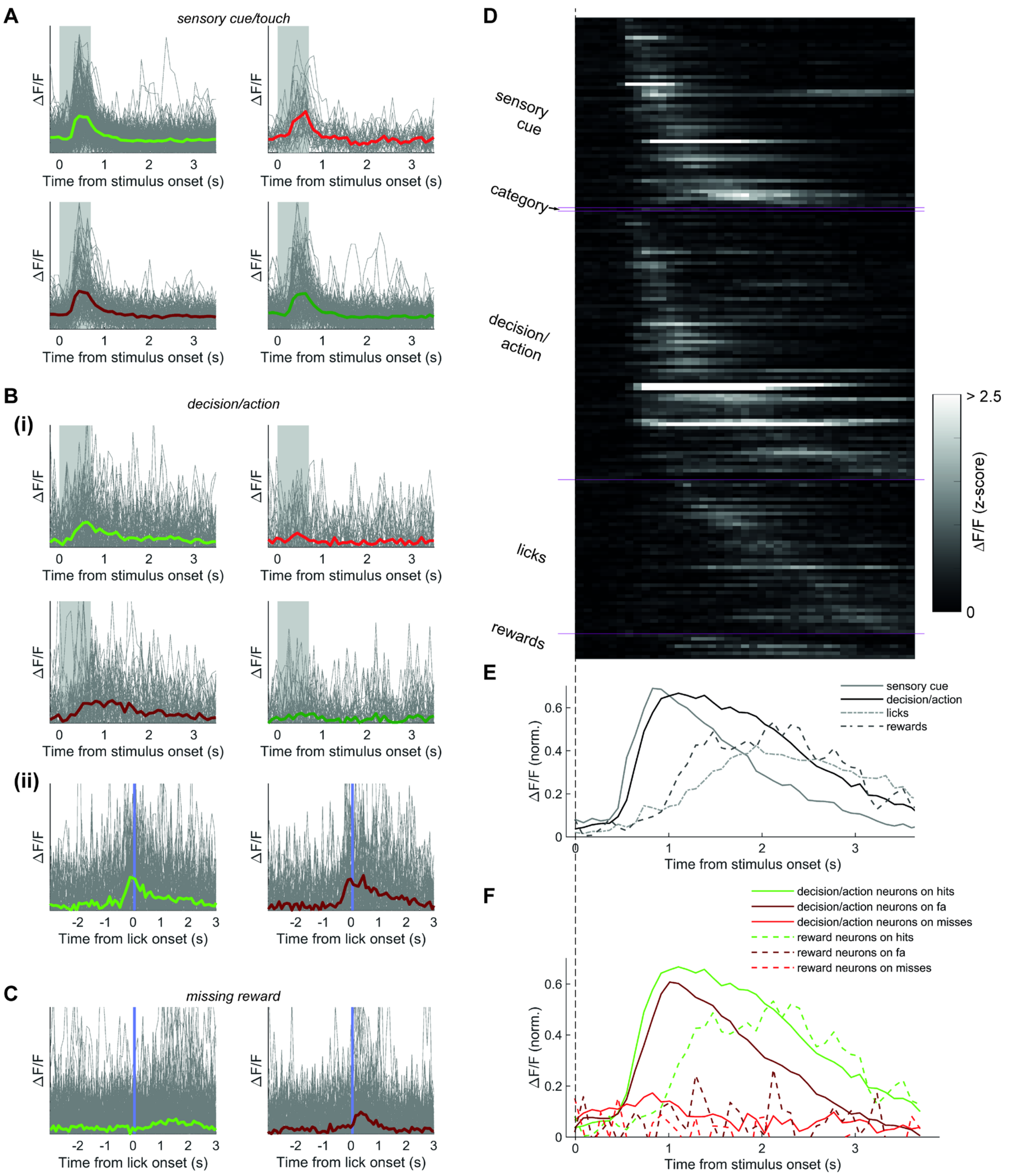

Figure S3. Dynamics and response properties of heterogeneous neurons in well-trained S1bf; related to Figure 3. (A) Trial-by-trial responses of the example 'sensory cue/touch' neuron from Figure 3D. $\Delta F/F_0$ relative to stimulation onset time (shaded region: stimulus presentation period). Thin gray lines, individual trials; thick line, mean. Each panel shows trials of a given type, indicated by the colour of the mean response (bright green, hits; bright red, misses; dark green, correct rejections; dark red, false alarms). Note how, on individual trials, $\Delta F/F_0$ peaks occur predominantly during stimulation, as befits a neuron responding to sensory input. **(B)** Trial-by-trial responses of the 'decision/action' neuron from Figure 3E. Colours as in (A). (i) Responses for all trial types timed relative to stimulus onset. Note how, on individual trials, $\Delta F/F_0$ peaks are spread throughout the trial, as befits a neuron whose responses relate to licks.

(ct'd from previous) (ii) Responses for trials with a lick response (hits and false alarms), timed relative to the onset of the first lick in the 'response period' (i.e. after the end of stimulation) (blue line). Single-trial peaks are seen to cluster around the time of the lick more than around any particular sensory stimulation cue and this gives rise to sharper peaks relative to lick time than stimulus time [compare with plots in (i)]. However, $\Delta F/F_0$ peaks occur throughout the trial, as expected for a neuron whose responses relate to licks. **(C)** Trial-by-trial responses of the 'missing reward' neuron from Figure 3G. Responses for trials with a lick response (hits and false alarms) timed relative to the onset of the first lick in the 'response period'. Colours as in (A). On individual trials, $\Delta F/F_0$ peaks can be found throughout the trial. However, on false alarm trials (right panel), peaks are concentrated just after the time of the first lick that would have been expected to generate a reward: of course, the reward is absent on false alarm trials and this evokes a response. In contrast, on hit trials, most peaks are spread over a much longer period beginning around 1 s after the first lick. This is explained because on any given hit trial, the animal will lick repeatedly until it uses up its water allocation for the trial, at which point the reward for licking will cease. This occurs at a variable time on each hit trial, hence the greater temporal spread of $\Delta F/F_0$ responses compared to the false alarm trials, and the smaller peak in the mean trace. **(D)** Heatmap of response dynamics for all visually classified neurons. Each row plots the mean response on hit trials for one neuron. Neurons are ordered according to their visually scored class (indicated on left), and then according to latency to peak. **(E)** Mean responses to hit trials as in (D), averaged across all neurons in each class. 'Sensory cue' neurons had the earliest response, followed by 'decision/action' neurons which predicted licks relevant to the task, then by 'rewards' neurons which reflected the outcome of task-relevant licks (i.e. reward presence or absence) and by 'lick' neurons which reflected licks regardless of context. **(F)** Comparison between the average responses of 'decision/action' and 'reward' neurons to hit, miss and false alarm trials. 'Decision/action' neurons responded prominently on hit and false alarm trials where the animal licked in response to the stimulus, but not on miss trials where the animal failed to lick to report the GO sequence; in contrast, 'reward' neurons responded prominently on hit trials where the animal licked and was rewarded, but not on miss or false alarm trials.
