# Supplementary material for: Sequence Learning Induces Selectivity to Multiple Task Parameters in Mouse Somatosensory Cortex
Source: Curr Biol. 2021 Feb 8;31(3):473–485.e5. doi: 10.1016/j.cub.2020.10.059 (PMC7883307; doi:10.1016/j.cub.2020.10.059)
Supplement: Document S2. Article plus Supplemental Information [file mmc2.pdf]

Article

Current Biology

Sequence Learning Induces Selectivity to Multiple Task Parameters in Mouse Somatosensory Cortex

Highlights

- Mice discriminate whisker motion sequences differing only in their elements' order
- Sensory input through somatosensory cortex is needed for sequence discrimination
- Barrel cortex neurons reflect variables including decision to lick and trial outcome
- Responses predicting licks appear as soon as mice associate a sequence with licking

Authors

Michael R. Bale, Malamati Bitzidou, Elena Giusto, Paul Kinghorn, Miguel Maravall

Correspondence

m.maravall@sussex.ac.uk

In Brief

Bale et al. analyze neuronal activity in layer 2/3 of somatosensory cortex as mice learn to discriminate between a target and a non-target ordered sequence of whisker motion. Neurons reflect task parameters including the learned association between target sequence and licking, rather than refining their tuning to sensory features.

Bale et al., 2021, Current Biology *31*, 473–485
February 8, 2021 © 2020 The Author(s). Published by Elsevier Inc.
https://doi.org/10.1016/j.cub.2020.10.059

Current Biology

CellPress
OPEN ACCESS

Article

Sequence Learning Induces Selectivity to Multiple Task Parameters in Mouse Somatosensory Cortex

Michael R. Bale,[1,2,4] Malamati Bitzidou,[1,3,4] Elena Giusto,[1] Paul Kinghorn,[1] and Miguel Maravall[1,5,*]
[1]Sussex Neuroscience, School of Life Sciences, University of Sussex, Brighton BN1 9QG, UK
[2]Present address: Scientifica, Uckfield, East Sussex TN22 1QQ, UK
[3]Present address: Neuroscience, The Francis Crick Institute, London NW1 1AT, UK
[4]These authors contributed equally
[5]Lead Contact
*Correspondence: m.maravall@sussex.ac.uk
https://doi.org/10.1016/j.cub.2020.10.059

SUMMARY

Sequential temporal ordering and patterning are key features of natural signals, used by the brain to decode stimuli and perceive them as sensory objects. To explore how cortical neuronal activity underpins sequence discrimination, we developed a task in which mice distinguished between tactile "word" sequences constructed from distinct vibrations delivered to the whiskers, assembled in different orders. Animals licked to report the presence of the target sequence. Mice could respond to the earliest possible cues allowing discrimination, effectively solving the task as a "detection of change" problem, but enhanced their performance when responding later. Optogenetic inactivation showed that the somatosensory cortex was necessary for sequence discrimination. Two-photon imaging in layer 2/3 of the primary somatosensory "barrel" cortex (S1bf) revealed that, in well-trained animals, neurons had heterogeneous selectivity to multiple task variables including not just sensory input but also the animal's action decision and the trial outcome (presence or absence of the predicted reward). Many neurons were activated preceding goal-directed licking, thus reflecting the animal's learned action in response to the target sequence; these neurons were found as soon as mice learned to associate the rewarded sequence with licking. In contrast, learning evoked smaller changes in sensory response tuning: neurons responding to stimulus features were found in naive mice, and training did not generate neurons with enhanced temporal integration or categorical responses. Therefore, in S1bf, sequence learning results in neurons whose activity reflects the learned association between target sequence and licking rather than a refined representation of sensory features.

INTRODUCTION

Natural sensory signals unfold over time, and their temporal patterning is inherent to their identity. Being sensitive to this patterning allows sensory systems to identify known stimuli, detect new or unexpected stimuli, and distinguish between objects. Thanks to this capacity, we can simultaneously recognize a favorite song playing on the radio and the identity of a family member from the cadence of their steps as they walk toward us. How is this ability underpinned by neuronal responses?

Within sensory modalities such as touch, the spiking responses of early sensory neurons faithfully relay temporally patterned signals to the brain for later integration and decoding.[1–10] Decoding such patterns could be facilitated by the known biophysical properties of central neurons and synapses. Neurons can become sensitive to specific spatiotemporal patterns of synaptic input,[11,12] *in vitro* networks of neurons can intrinsically encode temporal input sequences,[13,14] and synapses mediating thalamocortical input can have diverse temporal filtering properties.[15] However, how these capacities relate to sensory sequence learning in a living animal is unknown. How does neuronal activity *in vivo* distinguish between relevant sequences? When does a categorical representation of sequences arise from learning?

We used the mouse tactile whisker system to test neuronal codes underlying sequence discrimination. Mice can discriminate between sequential patterns of whisker vibration, reaching performance levels comparable to humans using their fingertips.[16] In this modality, sensory information from the whiskers first reaches the cortex through the "barrel field" of the primary somatosensory cortex (S1bf), the main cortical target for direct somatosensory input from the thalamus.[17] Neurons in S1bf encode well-defined stimulus features, but in naive animals, they do not integrate sensory information over time.[18–24] Here, we determined whether S1bf and successive cortical processing stages are needed to solve an elementary sequence discrimination task, and how the selectivity of neuronal responses in S1bf changes as a result of learning.

RESULTS

Discrimination of Elementary Tactile Sequences in Mice

We trained head-fixed mice to respond selectively to a target sequence of vibrations delivered to the whiskers (Figure 1). In

This is an open access article under the CC BY license (http://creativecommons.org/licenses/by/4.0/).

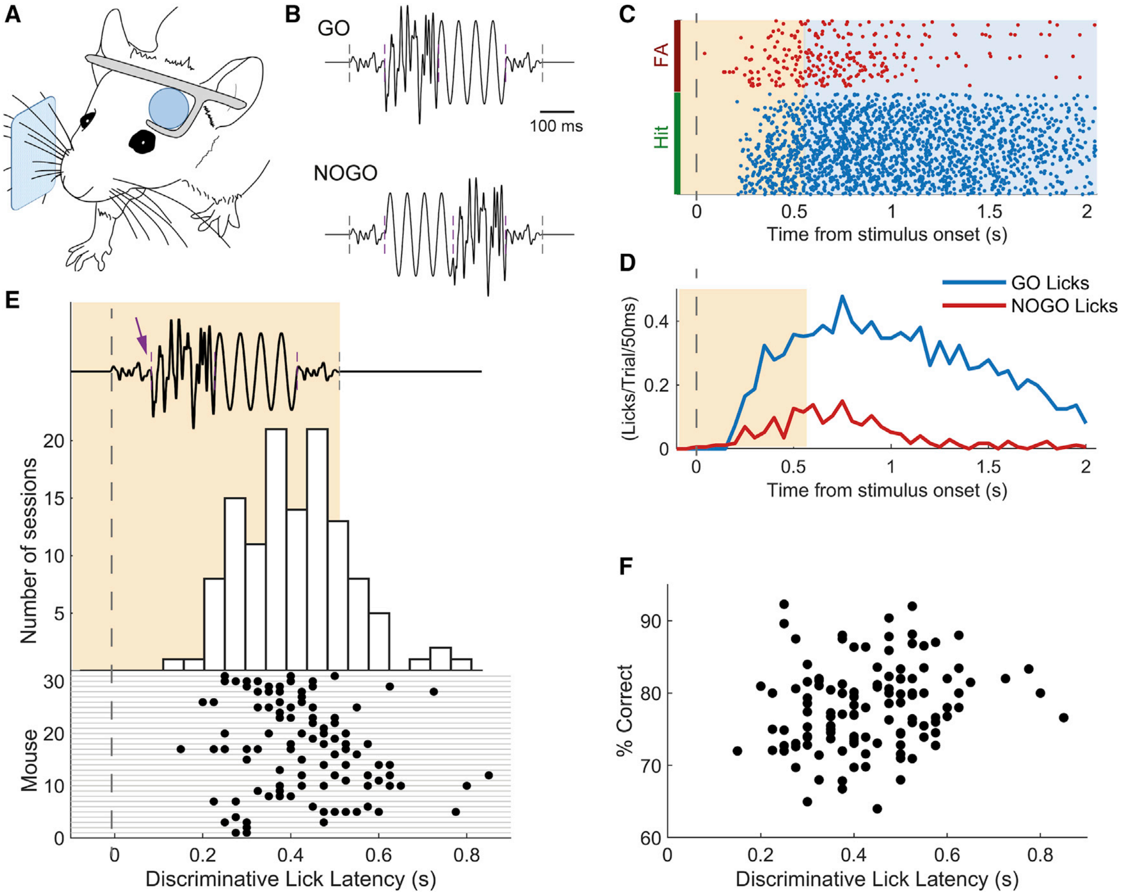

Figure 1. Discrimination of Elementary Tactile Sequences in Mice

(A) Diagram of experimental setup. Sensory stimulation was delivered to head-fixed mice via the whiskers.

(B) The GO and NOGO stimulus sequences consisted of four segments and differed in that the order of the central segments was switched. Gray dashed lines mark start and end of sequences; purple dashed lines, transitions between segments.

(C) Raster plot of licks on GO (hit) and NOGO (false alarm) trials for an example session. Shading shows stimulus presentation period.

(D) Histogram of licks on GO and NOGO trials for the same example session. The time at which traces diverge is termed the discriminative lick latency, DLL.

(E) Histogram (top) and raster plot (bottom) showing DLL over the course of a session. Raster displays data for all mice (each mouse, one row) and sessions (each session, one data point). Time is relative to start of stimulus sequence (top). Purple dashed lines, transitions between segments; arrow indicates time when target GO sequence (shown here) diverges from NOGO sequence and can first be distinguished.

(F) Performance (percent correct) plotted against discriminative lick latency across all sessions.

See also Figure S1.

this GO/NOGO discrimination design, target and non-target sequences differed in the order of their (initially meaningless) elements. Stimulation was delivered to multiple whiskers (STAR Methods).

Building on our earlier finding that mice learn to recognize tactile sequences constructed as a concatenation of noise segments,[16] we used a simple sequence design in which each stimulus consisted of a tactile “word” (Figure 1B). Each segment within the word comprised either filtered noise (with different amplitudes) or sinusoidal stimulation. Following a GO sequence, if mice licked during the response period (hit trial), they received a water reward; if they failed to lick (miss trial), the next trial began as normal. Following a NOGO sequence, if mice correctly withheld licking during the response period (correct rejection trial, CR), the next trial began as normal; if they licked (false alarm trial, FA), the next trial was delayed by 2–5 s, with the duration set depending on mouse thirst and impulsiveness.

After training, mice learned to associate one specific sequence with licking for a water reward (Figures 1C and 1D). We measured performance as percentage correct, computed using the correct number of trials divided by the total number, measured over a 50-trial sliding window (corrected for the proportion of GO and NOGO trials; STAR Methods). Animals took 2–26 training sessions to reach 70% correct discrimination (median 5 sessions, interquartile range 3–7 sessions). Mice achieved a mean of 80% performance in their best-performing session (SD 7%, n = 42 animals).

What cues did mice use to distinguish the learned sequence? An ideal observer would be able to distinguish the identity of a sequence immediately upon the first transition between its constituent segments, as this was the moment at which GO (target)

and NOGO (non-target) sequences diverged (Figure 1B). In our design, mice were allowed to lick during the sequence presentation period without incurring reward or punishment (STAR Methods), and this allowed us to measure their freely varying response (lick) times. For each session, we determined the time at which lick rates on GO (hit) trials diverged significantly from those on NOGO (FA) trials, usually well before the end of stimulation (Figure 1D). This time gave an upper-bound estimate for when the mouse, on average, reached its decision as to sequence identity in that session (STAR Methods). We term this measure ''discriminative lick latency'' (DLL).

Across a dataset of 31 mice and 122 sessions, DLL varied by animal and session (Figure 1E). The DLL was sometimes short enough to suggest the animal made its decision immediately upon detecting the earliest possible cue distinguishing the GO from the NOGO sequence, at the transition upon the end of the shared initial segment (100 ms from stimulation onset; Figure 1B). However, in other sessions, the value of the DLL suggested longer deliberation (range 150–850 ms, median 425 ms).

Either strategy—instantaneous response or deliberation—could potentially lead to high performance, depending on conditions. An ideal instantaneous detector, under noiseless conditions with no variability across stimulus presentations, would be able to identify the target immediately upon the first transition (Figure 1E, arrow): in this scenario, slower responses would imply no gain in performance and might even be a signature of impaired performance in a poor learner. This could be reflected in an absence of correlation between DLL and performance, or in longer DLLs corresponding to lower performance. On the other hand, under real-life conditions, one would expect variability in whisker stimulation from trial to trial: the stimulator might interact differently with the whiskers, the animal might itself move the whiskers, or its attention might wander. Given this variability, on any given trial, the identity of the sequence could become clearer over time; thus, it could be beneficial for mice to have the capacity to integrate sensory information for longer in order to do better. This is because the accumulated difference between the GO and NOGO sequences grew over time from the first transition, potentially making it easier for a mechanism of evidence integration to detect the identity of the sequence as time went on. In this situation, longer DLLs would correspond to higher performance. Our results are consistent with the latter scenario: sessions with greater integration or deliberation, as measured by a longer DLL, correlated with higher performance (Figure 1F; 122 sessions, $t = 2.45$, $p = 0.016$, mixed-effects model with mouse ID as random factor). We did not find support for the alternative possibility that mice with higher performance had longer DLL simply because they learned to defer licking for their reward (STAR Methods).

To visualize the variation in DLL across sessions and mice, we plotted the full set of DLLs for each animal (bottom raster in Figure 1E). This showed that different mice varied in their tendency to accumulate information versus making quick decisions, but that differences across mice did not account for the majority of the variation in the dataset, as there was considerable variability in DLL across sessions within each mouse. Variation within mice accounted for approximately 65% of the total variance in DLL, with the remaining 35% occurring across mice (sum of squares calculations).

These results demonstrate that mice readily learned to associate a specific tactile sequence with a water reward. Animals were often able to discriminate quickly, consistent with an ability to focus on the earliest cues that allowed discrimination. However, performance on the task tended to be higher when animals took longer.

Somatosensory Cortex Carries Sensory Information Needed for Sequence Discrimination

To track the flow of activity through early cortical stages during task performance and determine which stages were needed for sequence discrimination, we trained mice expressing channelrhodopsin in cortical GABAergic interneurons (VGAT-ChR2-EYFP[25]). Once mice had achieved 75% correct performance during a session, we began running sessions combining optogenetics with behavior. We suppressed activity in stereotaxically defined regions of dorsal cortex throughout stimulus presentation (from 50 ms before onset to 50 ms after offset), illuminating the cortical surface with a blue laser (STAR Methods). Laser-ON and laser-OFF trials were interspersed, with laser-ON comprising a randomly chosen subset (20%) of trials. In addition to S1bf, we selected the following regions for optogenetic suppression, all of which receive direct projections from S1bf: secondary somatosensory cortex (S2), posterior parietal cortex (PPC), and whisker primary motor cortex (wM1). S1bf and S2 are part of the ascending cortical pathway for tactile input. PPC is a center for multisensory sensorimotor integration and its activity has been shown to accumulate sensory evidence over time and reflect history biases.[26–28] M1 is a center regulating the learning and deployment of relevant motor responses, but can also accumulate sensory evidence over time[29] and acts as a goal-directed modulator or inhibitor, rather than just a generator, of motor actions.[30]

Optogenetic suppression centered over either S1bf or S2 significantly decreased lick response rates (the percentage of trials with a lick response; Figures 2B and 2C) (S1bf: 4 mice, 10 sessions, $F[1,36] = 97.8$, $p < 10^{-11}$; S2: 3 mice, 11 sessions, $F[1,40] = 308$, $p < 10^{-19}$; both two-way ANOVA). This decrease in response rate affected both GO and NOGO trials (Figures 2B and 2C), but was greater on GO trials (S1bf: $F[1,36] = 7.40$, $p = 0.01$; S2: $F[1,40] = 45.7$, $p < 10^{-7}$). The fact that the decrease in response rate occurred both on GO and NOGO trials indicated that optogenetic manipulation was not disturbing a specific representation of the GO target sequence; rather, it likely interfered with the overall flow of sensory information through somatosensory cortex.

We also considered the alternative possibility that S1bf/S2 inactivation did not suppress sensory information needed for the decision, but caused a nonspecific decrease in the probability or speed of elicited motor actions, leading to the reduced response rate. If manifested as an overall scaling down of licking probability, such an effect would still result in a lower rate of FA trials than hits. However, on laser-ON trials, the resulting FA rate was no lower than the hit rate. Indeed, there was no significant difference between the probability of responding to GO and NOGO stimuli so that discrimination fell to random levels (Figures 2B and 2C; d' upon laser stimulation did not differ significantly from 0: S1bf-centered inactivation, $p = 0.967$; S2-centered, $p = 0.880$; both Wilcoxon signed rank). This implies that licking

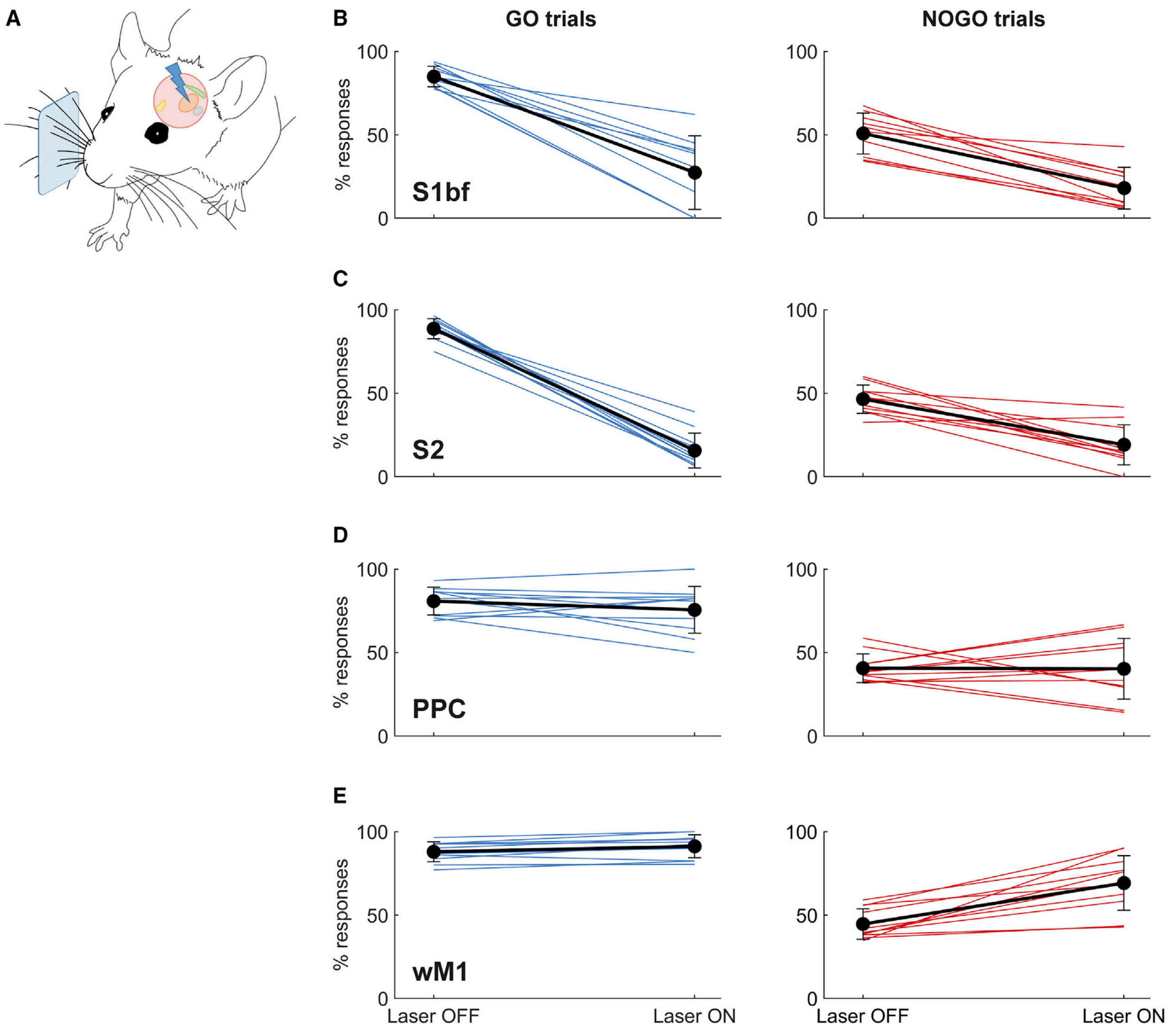

Figure 2. Tracking the Participation of Different Cortical Regions with Optogenetic Activity Suppression

(A) Diagram of experimental setup. Cortical areas were illuminated with a blue laser during trial performance.

(B) Effects of S1bf suppression on performance (lick response rates) on GO (left) and NOGO trials (right).

(C) Effects of S2 suppression.

(D) Effects of PPC suppression.

(E) Effects of wM1 suppression. Black lines: means across sessions; error bars: SD; colored lines: individual sessions.

See also Figure S2.

probability was more strongly reduced by optogenetic inactivation on GO trials, and therefore inactivation did not simply produce an overall scaling down of licking probability regardless of trial type. Moreover, on laser-ON trials, there was no evidence for lick responses becoming slower: median latency to first lick was no different on laser-ON and laser-OFF trials (S1bf-centered inactivation, $p = 0.578$; S2-centered, $p = 0.365$; both Wilcoxon signed rank). Therefore, our findings cannot be explained by a nonspecific decrease in the rate or speed of licking.

These results indicate that the sensory signals necessary for sequence discrimination were routed through S1bf and S2. Suppression centered over either area had a similar effect, consistent with S1bf-S2 serial flow of sensory information or, alternatively, with an S1bf-S2 loop activated in series.[31–35]

Suppressing PPC had no systematic effect on response rate (Figure 2D) (3 mice, 11 sessions, $F[1,40] = 0.52$, $p = 0.475$, two-way ANOVA). Suppressing wM1 disinhibited lick responses, particularly on NOGO trials, i.e., those in which mice had been trained selectively to avoid licking (Figure 2E) (3 mice, 11 sessions, $F[1,40] = 19.7$, $p < 10^{-4}$, two-way ANOVA; interaction between trial type and effect of laser, $F[1,40] = 11.5$, $p = 0.0016$). Disinhibition of lick responses was significant on NOGO ($p < 10^{-4}$, Tukey-Kramer) but not GO trials ($p = 0.880$, Tukey-Kramer). This is consistent with earlier studies showing that

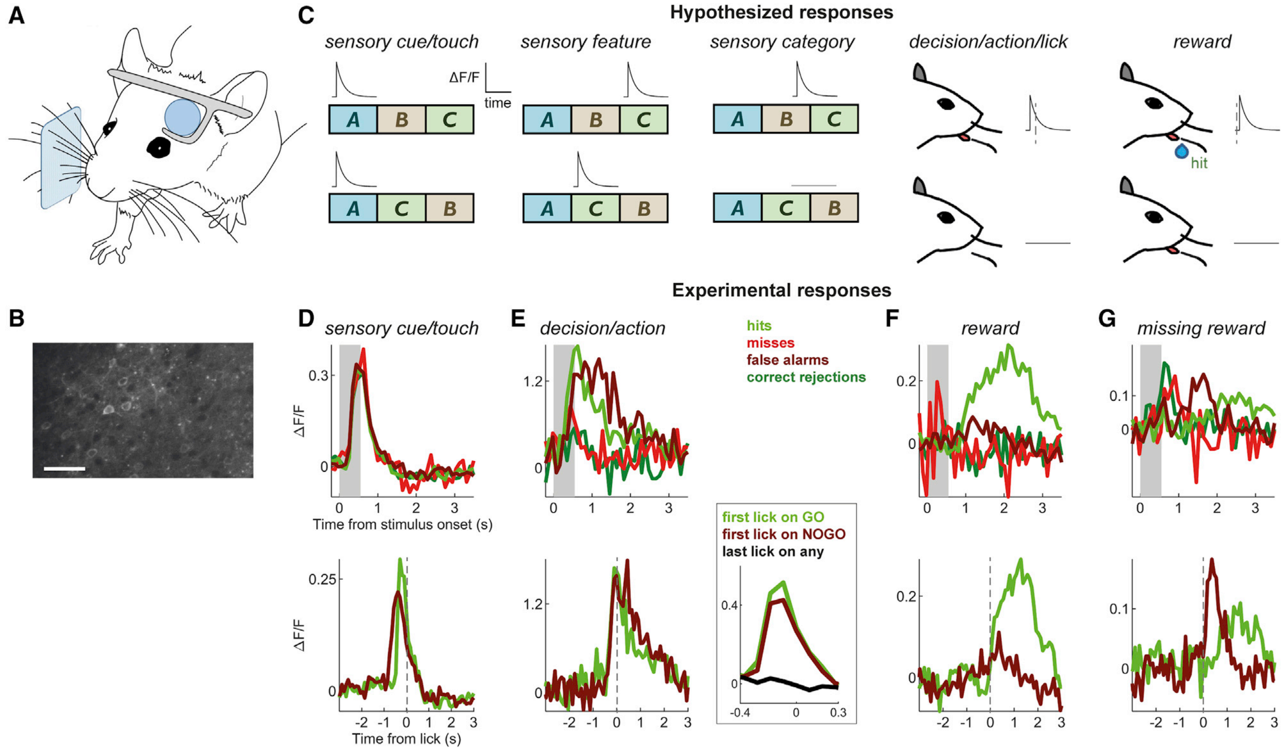

Figure 3. Neuronal Responses in Well-Trained S1bf Reflect Heterogeneous Task Variables and Learned Associations

(A) Diagram of experimental setup. Two-photon imaging was carried out while animals performed the task.

(B) Example of field of view (scale bar, 100 μm).

(C) Hypothetical expected responses to presentation of a target sensory sequence (ABC) and a sequence whose segments' order has been scrambled (ACB). Diagrams show the $\Delta F/F_0$ responses presented by hypothetical neurons reflecting one of the following: sensory cue presentation onset, a specific sensory feature (segment C in the example), sensory category (ABC versus ACB), the prediction of the decision to lick, or the presence of a reward. In the last two cases, note the different timing of the response relative to the lick (dashed line).

(D–G) Actual experimental responses. (D) Example neuron responsive to sensory cue. Top, mean $\Delta F/F_0$ relative to stimulation time (shaded region: stimulus presentation period). Bottom, mean $\Delta F/F_0$ relative to first lick (dashed line). (E) Example neurons predictive of licking. Left panels, one neuron; right panel, different neuron highlighting the difference between responses to first and last licks. Note that peak response precedes licking for both neurons. (F) Example neuron responsive to reward on hit trials. Note that peak only builds up after the end of stimulation and once the lick is over. (G) Example neuron responsive to the absence of a reward on false alarm trials.

See also Figure S3.

optogenetic suppression of M1 can increase FA rates[30] and with the notion that M1 activity can regulate the learned modulation or suppression of a motor action, rather than always positively eliciting actions.[36,37] Overall, these findings show that the sensory information necessary for sequence discrimination was carried by somatosensory cortex but not PPC or wM1.

Neuronal Responses in Well-Trained S1bf Reflect Heterogeneous Task Variables and Learned Associations

To investigate neuronal responses in primary sensory cortex during sequence discrimination, we used two-photon imaging in layer 2/3 (Figure 3). Mice were Thy1-GCaMP6f animals expressing calcium indicator in cortical excitatory neurons[38] (STAR Methods; 7 mice, 27 sessions). For each neuron, we extracted the differential fluorescence ($\Delta F/F_0$) time series on every trial and computed the average $\Delta F/F_0$ response profile parsed by trial outcome (hit, miss, FA, CR), using the beginning of the trial as temporal reference. We also computed the average $\Delta F/F_0$ response relative to the time of the first lick on hit and FA trials. These visualizations allowed us to explore the relationship between trial type and changes in fluorescence (Figures 3C–3G).

Based on the notion that S1bf primarily provides sensory information to higher stages in a behavioral hierarchy for sensory-guided decision making, we expected our data to be dominated by neurons representing features of sensory stimulation sequences (Figure 3C), potentially including neurons categorically selective to the overall identity of the learned sequence (in the figure, these are "sensory category" neurons responding only on GO trials).

In the event, neurons in S1bf of well-trained mice showed heterogeneous selectivity to multiple task variables including the onset of sensory input ("sensory cue/touch" neurons, Figures 3C and 3D), the animal's decision to act with a lick ("decision/action," Figures 3C and 3E), and the subsequent outcome of the trial including the presence or absence of expected rewards ("reward," Figures 3C, 3F, and 3G). We placed neurons into one of several mutually exclusive classes, depending on their

Table 1. Proportions of Visually Identified S1bf Layer 2/3 Neurons Responding to Sensory and Task Variables in Well-Trained and Naive Mice

	Class of Neuronal Response							
	Sensory Cue	Sensory Feature Selective	Sensory Category	Decision/Action Predictive (during Stimulus Period)	Decision/Action Predictive (during Response Period)	Licking	Reward Delivery and Error	Unclassified
Well-trained	53 (17%)	0	1 (0.32%)	70 (22%)	5 (1.6%)	43 (14%)	7 (2.2%)	136 (43%)
Naive	23 (6.5%)	22 (6.2%)	2 (0.56%)	7 (2%)	4 (1.1%)	4 (1.1%)	10 (2.8%)	284 (80%)

responses to sensory and other variables (Table 1). The most frequently found classes were sensory cue/touch neurons that responded to the onset of stimulation regardless of trial type (Figures 3D and S3A) and decision/action cells activated just before goal-directed licking, the animal's learned response to the target sequence (Figures 3E and S3B).

Sensory cue/touch neurons responded non-specifically to sensory stimulation. Because GO and NOGO sequences shared a common initial segment, a neuron sensitive to stimulus onset and with strongly adapting responses would "view" the sequences as identical; many layer 2/3 neurons labeled as "sensory cue" in the present task would likely appear as touch-sensitive neurons in situations where an animal encounters objects with its whiskers.[39–41]

Decision/action predictive neurons were more strongly active on hit and FA trials than on miss or CR trials, so their activity was better modulated by the animal's motor response than by the identity of the sequence (Figures 3E, S3B, and S3F). Typical decision/action responses were temporally locked to licking rather than to stimulus presentation, and preceded licking (Figure 3E). For example, the neuron in Figure 3E (left) had a response latency (determined as the time taken for $\Delta F/F_0$ to depart more than 3SD from its mean baseline) of −290 ms relative to lick time on hit trials. In these neurons, responses preceding licks were not stereotypical, but differed for early and late licks in a trial, reflecting the licks' relevance during task engagement (Figure 3E, right inset). This differentiated such action-predictive neurons from a rarer class that appeared purely to reflect the motor action of a lick, regardless of context (termed "licking" in Table 1). Neurons in this smaller licking class encoded licking movements independent of task learning.

Finally, some neurons displayed readily identifiable preferences for trial-to-trial task outcome variables, including an expected reward's delivery (Figure 3F) or absence (Figure 3G). Consequently, these neurons were selectively active on hit versus FA trials when responsive to the presence of the reward (Figures 3F and S3F) or on FA versus hit trials when responsive to the absence of the expected reward (Figures 3G and S3C). A hallmark of these responses was that they followed licks and their outcomes rather than preceded them.

Overall, across our dataset of S1bf neurons in well-trained mice (n = 315 neurons), n = 179 (57%) were visually classifiable according to the classes above (Table 1). Those not classified included neurons with no activity, as well as those with activity not visually related to any of the variables monitored. Within the classifiable neurons, those with a pure sensory response were in the minority (n = 54; 30% of classified, 17% overall). Moreover, only one neuron was identifiable as providing pure sensory categorical encoding of trial type (i.e., responding to either the GO or NOGO sequence in a manner independent of the animal's behavior, as in the sensory category hypothetical example in Figure 3C). In contrast, we frequently observed action-predictive neurons whose responses reflected the learned association between a stimulus perceived as the target sequence, and the consequent action (n = 75; 42% of classified, 24% overall).

Diverse Neuronal Response Classes in Well-Trained S1bf

The visual classification described above suggested that S1bf neurons in well-trained animals behaved heterogeneously. To assess this quantitatively, we analyzed the extent to which different cells preferentially responded with distinct patterns or profiles (as in Figures 3C–3G). If response profiles during a trial fell into distinct subsets, neurons within each subset would be expected to have greater response similarity than expected if their profiles varied at random. To test this, we performed a projection angle index of response similarity (PAIRS) analysis (STAR Methods).[42] We found that the median response similarity between neurons in our dataset was greater than for any of 10,000 random surrogate neuronal datasets (Figure 4A; $p < 10^{-4}$). Thus, PAIRS analysis established that neuronal responses clustered more than expected by chance.

This result, taken together with the observation that neuronal responses reflected distinguishable aspects of the task (Figures 3D–3G), suggested the existence of neurons with distinct functional properties. We thus developed a classifier analysis to quantify whether a neuron conveyed information about sensory trial type (GO versus NOGO) or the animal's response (lick versus no-lick), based on how the neuron's response evolved during a trial (STAR Methods). This analysis showed that 25% of neurons (70 out of n = 277) were able to support classification of sensory trial type (GO versus NOGO) to criterion level (Figure 4B; defined as the neuron performing better than 95% of surrogate classifiers constructed by shuffling trial labels). Note that this proportion does not include all neurons responsive to stimulation: for example, a sensory cue neuron (e.g., Figure 3D) would not be able to classify GO versus NOGO, as it would respond very similarly on both types of trial. In terms of classifying whether the mouse response on a trial was lick versus no-lick, 30% of neurons (86 out of n = 284) could perform to criterion level (Figure 4B).

Figure 4B plots classification performance on lick versus no-lick against performance on GO versus NOGO, shown for all neurons for which both classifiers could be computed (n = 272). Across neurons, classification performance was higher

CellPress
OPEN ACCESS

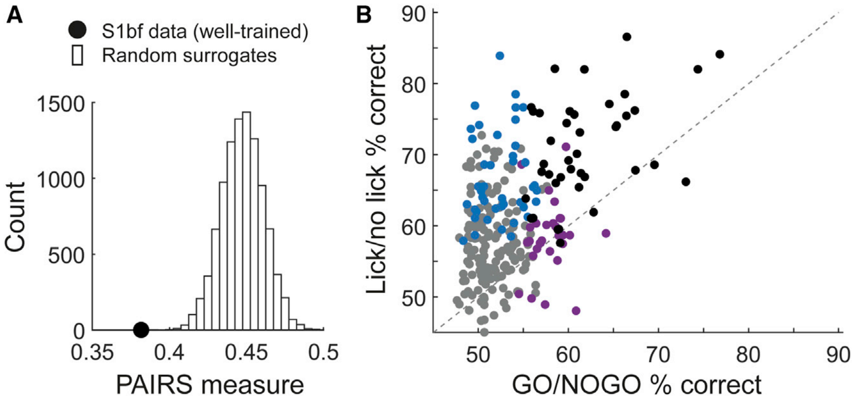

Figure 4. Diverse Response Classes in Well-Trained S1bf

(A) Median PAIRS value (index of response similarity) for experimental S1bf dataset compared to distribution for 10,000 random surrogates. The response properties of experimental neurons clustered more than expected by chance.

(B) Classification performance (percent correct) supported by individual neurons. Data points shows lick versus no-lick performance plotted against GO versus NOGO performance for each neuron. Black dots: neurons with significant performance on both lick versus no lick and GO versus NOGO. Blue: neurons with significant performance on lick versus no-lick. Purple: neurons with significant performance on GO versus NOGO. Grey: neurons with significant performance on neither. Dashed line: equality.

for lick versus no-lick than GO versus NOGO ($p < 10^{-34}$, Wilcoxon signed rank). Notably, multiple neurons supported lick versus no-lick classification with high performance (70% or above), consistent with our identification of action-predictive neurons in the observations above (Figure 3; Table 1). While some neurons could discriminate GO versus NOGO sequences with good performance, rarely did they do so without also showing sensitivity to the upcoming lick action. In other words, sensory representations in individual S1bf neurons of well-trained animals were tangled with, and not independent from, action representations. This is a striking result considering that S1bf is a textbook sensory area and deviates from the expectation that sensory responses would dominate our dataset.

Neuronal Responses in S1bf Selective to the Target Sequence and Associated Actions Appear as Soon as the Association Is Learned

Given that a salient feature of our well-trained S1bf data was the presence of neurons whose activity predicted licking to the learned sequence or was sensitive to trial outcome (Figure 3), we sought to determine when these responses arose during task learning. We therefore repeated the above procedures in animals that had been trained to remain head fixed but not yet to detect or discriminate a sequence stimulus (4 mice, 14 sessions; Figure 5). Imaging spanned from the first training session to the fourth. Mice began to lick preferentially to the GO stimulus starting in the second session.

We found that neurons in these naive animals participated more sparsely in task encoding: a smaller proportion than in well-trained mice could be classified into the classes defined earlier (Table 1; 20% of 356 neurons versus 57% of 315 neurons; $p < 10^{-22}$, odds ratio 5.19, Fisher's exact test). Strikingly, some neurons in naive mice could be identified as selective to sensory features; i.e., they responded selectively to specific segments of the sequence (Figure 5A). However, in the first training session, we found no action-predictive neurons: contrary to well-trained animals, no neurons showed responses time locked to licking (e.g., Figure 5A). Thus, training increased the proportion of neurons visually responsive to sensory and, particularly, action-related variables.

PAIRS analysis showed that, just as in well-trained animals, S1bf neurons in naive mice also had clustered response profiles (Figure 5B; $p < 10^{-4}$). Accordingly, we again performed a classifier analysis to quantify the fraction of neurons that could support classification of trial identity (GO versus NOGO) or licking response (lick versus no-lick). The ability of neurons to classify trial type progressed over the course of training (Figure 5C). For neurons imaged in the first training session, 12% (11 out of n = 89) could support GO versus NOGO classification. In naive animals overall, 14% of neurons (46 out of n = 318) could classify GO versus NOGO trial type; this fraction was significantly lower than in well-trained animals (25%; p = 0.0081, odds ratio 1.75, Fisher's exact test), consistent with training inducing an increase in the proportion of neurons representing trial type (i.e., a decrease in population sparseness).

Classification performance on lick versus no-lick trials progressed in a different manner: in the first training session, we found no neurons that could support lick versus no-lick classification (Figure 5C). This changed from the second session: 22% of neurons imaged in sessions 2–4 (39 out of n = 176) could classify trials as lick versus no-lick, a percentage not significantly different from that in well-trained animals (30%; p = 0.174, odds ratio 0.732, Fisher's exact test). Thus, neurons whose activity differed significantly on lick versus no-lick trials were first found on the second day of training, at the same time as animals first associated the target sequence with licking for a reward and the non-target sequence with suppression of licking.

The classification performance of significant "lick versus no lick" neurons increased during training (Figure 5D; n = 39 for sessions 2–4, n = 86 for well-trained, p = 0.00019, Wilcoxon rank sum). In contrast, although the proportion of significant "GO versus NOGO" neurons increased with training (Figure 5C), their classification performance did not (Figure 5E; n = 46 for sessions 1–4, n = 70 for well-trained, p = 0.168, Wilcoxon rank sum). Thus, neurons whose activity reflected sensory trial type did not refine their selectivity with training.

These results demonstrate that, as a result of the explicit learning of a target sequence of whisker stimulation, neurons in somatosensory cortex reflect multiple task variables, and particularly the learned association between sensory sequence and goal-directed decision to act.

DISCUSSION

Training mice on a whisker-mediated sequence discrimination task, we found three main results: (1) animals appear to solve

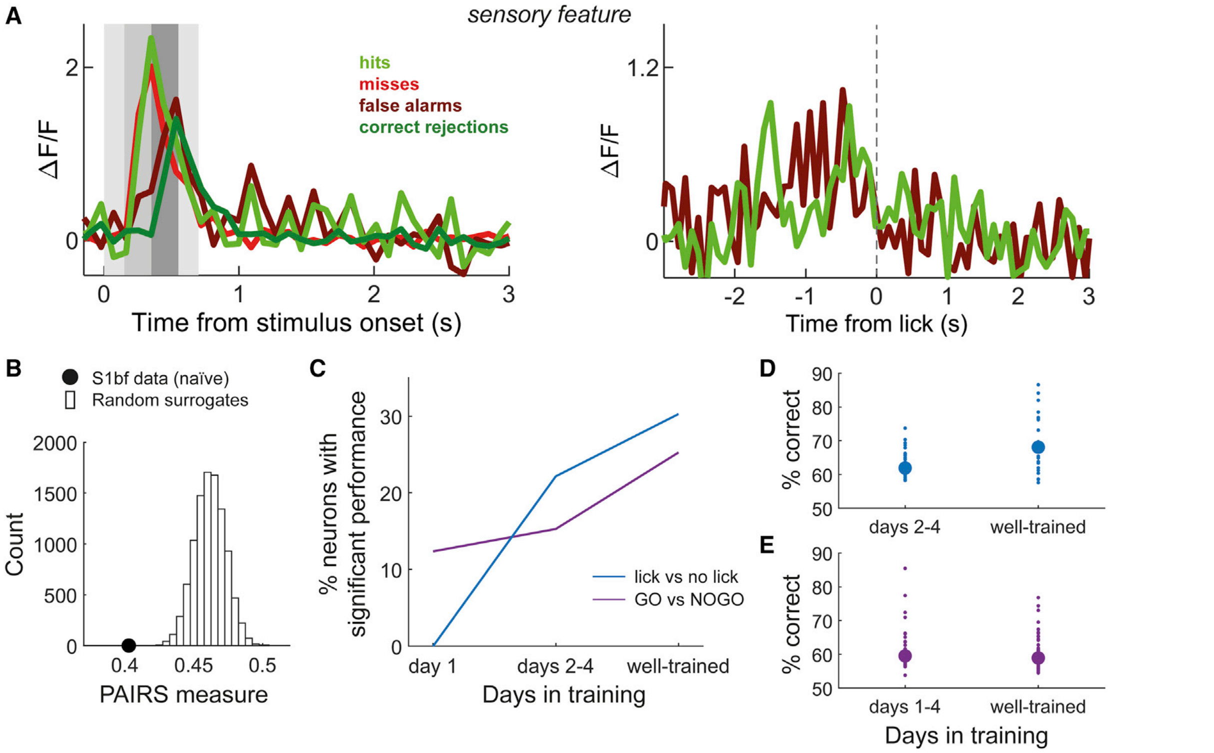

Figure 5. Progression of Neuronal Selectivity to Sequence Identity and the Associated Actions over the Course of Training

(A) Example neuron selective to a specific sensory feature. Left, mean $\Delta F/F_0$ relative to stimulation time. Striped background demarcates segments in the stimulus sequence. Note how the neuron responded later on NOGO trials (FA and CR) than on GO trials (hits and misses), indicating sensitivity to sensory features present at different times depending on trial type and regardless of the animal's action (hit and miss responses were similar to each other, and so were FAs and CRs). Right, mean $\Delta F/F_0$ relative to first lick (dashed line). The neuron's average response was not time locked to licking.

(B) Median PAIRS value (index of response similarity) for naive S1bf data compared to distribution for 10,000 random surrogates.

(C) Progression of the percentage of neurons with significant performance with days in training. Progression is significant for both curves: for GO versus NOGO classification, χ^2 statistic = 11.36, p = 0.0034, χ^2 test. For lick versus no-lick, χ^2 statistic = 9.97, p = 0.0068, χ^2 test.

(D) Classification performance for significant neurons on lick versus no-lick for different stages of training. Small dots: individual neurons. Thick dot: median.

(E) Classification performance for significant neurons on GO versus NOGO for different stages of training. Small dots: individual neurons. Thick dot: median.

the task by seeking the earliest cues that predict the identity of the target sequence, but perform better when deliberating and responding later; (2) somatosensory cortex is needed for performing the task; and (3) neurons in superficial layers of S1bf display heterogeneous selectivity to task variables including sensory input, the animal's action decision, and trial outcome—with the latter selectivity being acquired as a result of learning to associate the target sequence with the action needed for reward. In naive animals, presenting a target sequence activates neurons selective to sensory input; upon training, presenting the same (rewarded) target sequence activates neurons that now, in large numbers, predict the animal's learned licking response. These results defied our expectation that neuronal activity in S1bf of well-trained animals would primarily represent features of sensory input, perhaps refined by learning; rather, we found neurons that embody learned associations between a sequence and the corresponding behavior and predict expected actions and outcomes in the context of the task.

Somatosensory Cortex, but Not PPC, Participates in Goal-Directed Tactile Sequence Discrimination

Our optogenetic experiments showed that suppressing activity in S1bf and S2 interferes with whisker-mediated sequence discrimination in a manner consistent with an interruption in sensory information. This indicates that somatosensory cortex is needed to perform the task. Similar conclusions have been reached for other tasks that demand recognition of whisker input streams.[34,43–47] In contrast, S1bf has been shown to be unnecessary for simple tasks involving detection of whisker motion.[48,49] While sensory cortex is not required for stimulus detection or localization, it may be needed for organizing sensory data into objects or concepts according to previous experience, including context-dependent change detection.[50,51] The effect of suppression centered over S1bf and S2 was specific to somatosensory cortex. Interfering with PPC activity had no discernible effect on task performance; under our task design, no comparisons of temporally separate stimuli were required, and the stimulus sequence did not need to be kept in working memory.

Plasticity Elicited by Goal-Directed Learning of a Sequence Couples Sensory Neurons to Action Decisions

Neurons in naive S1bf have limited temporal integration: they report on sensory input accumulated over just a few tens of milliseconds.[18–23] In earlier studies, this temporal integration remained limited even after learning tasks in which performance could benefit from greater integration.[22] This property of being responsive to sensory signals that are current rather than accumulated over time is shared with neurons in the primate primary somatosensory cortex,[52] suggesting a common principle across mammalian tactile pathways.

Neurons can intrinsically discriminate between stimulus sequences, and modeling studies have shown that generally observed forms of synaptic plasticity can endow neurons with sensitivity to input patterns lasting longer than the neuron's membrane integration timescale.[53–55] Our expectation was therefore that conditioning on a specific sequence might produce S1bf neurons that preferentially represented that sequence, now habitually present in the animal's life and associated with a desirable goal. This would fit in with a framework in which the role of sensory cortex is primarily to provide sensory representations,[56] and learning refines these representations to become more predictive of upcoming sensory signals or increasingly modulated by behavior.[57] Instead, we found neurons that—upon training but not at the outset—directly embodied the association between the target sequence and the appropriate learned response, but did not respond selectively to the sequence independently of the animal's response. We suggest that these neurons may have become predictive of the learned outcomes associated with sensory stimuli to which they were originally tuned.[51] Note that even after learning, a considerable number of neurons responded simply to stimulus onset (Figures 3C and 3D) and showed no modulation related to licking; thus, our results are inconsistent with sensory signals being occluded by a spread of prevalent lick-related activity.

Non-sensory Representations in Sensory Cortex

Classic accounts of perception posit a serial feedforward scheme whereby successive processing steps map onto neuronal activity elicited in distinct brain regions, with each region's responses classifiable as essentially sensory-, decision-, or action-related. These conclusions were originally derived from classic experiments carried out under anesthesia; in this condition, neurons in primary sensory cortex respond to specific physical features of stimuli, while neurons in higher areas such as the prefrontal cortex (PFC) cannot be characterized as sensory feature selective. More recent experiments have often involved training animals to produce a sensory-guided response only after the period of sensory stimulation has concluded. This temporal separation between stimuli and action readily allows for isolation of neuronal activity reflecting different aspects and stages of the trained behavior and has produced deep insights into how these map onto neuronal processing stages (reviewed in Romo and de Lafuente[52]).

Conversely, in situations where an animal is permitted to act while an ongoing stimulus is being presented, a complex interplay occurs between brain regions, and neuronal activity propagates in an intricate pattern, reflecting many aspects of the task. Neurons in primary sensory cortex respond to multiple task variables, including not just sensory signals, but also the animal's decision on whether to act and the decision's outcome. This rich pattern of activity is likely to represent the neuronal substrate for the animal's interactions with a dynamic environment as often played out in real life, where sensory signals and our responses to them are ongoing and intertwined in time.

Although inconsistent with a classic feedforward picture of cortical processing, our data are consistent with multiple recent findings in other contexts. In those studies, variables affecting neurons in sensory cortex include the decision to act and choice of action, arousal and attention, spontaneous gestures and motions, mismatches between actual and expected sensory input, motor activation relevant to task execution, and expected and actual rewards and their timing.[34,35,58–85] In our data, non-sensory parameters do not just modulate the responses of sensory cortex neurons; rather, in a subset of neurons, responses reflect action decisions or reward outcomes more strongly than sensory cues or features, consistent with other recent studies.[79,86,87]

Sensitivity to multiple sensory and behavioral variables is well established in higher cortical areas such as PFC[88–92] and parietal cortex.[42,93] The findings above suggest that, upon learning, a similarly rich representation becomes shared by early sensory areas. This could happen through top-down connections[94,95] broadcasting a "copy" or version of frontal cortex responses to sensory cortex. Supporting this idea, non-sensory activity reflecting motor actions spreads widely across dorsal cortex when an animal is engaged in a learned task,[83,96–100] perhaps as an "efferent copy" signal;[101] this broadcast seems to originate in a frontal premotor region linked to action selection and motor preparation.[47,96–98,100,102] In rodents, the somatosensory cortex links directly to PFC, premotor cortex, and the basal ganglia (dorsal striatum), areas which have been linked to accumulation of sensory evidence, to categorization, and to reaching the decision itself;[26,31,52,100,102–105] learning an association between sensory information and specific actions is likely to reinforce feedback loops between sensory and higher regions.[33,34,74,86,98,106] The links between a stimulus and the appropriate action could be learned initially in one of the higher areas. Depending on behavioral context and task familiarity, top-down connections to S1bf and S2 could then help link the lower-level representation of the target stimulus to its consequent action. It will be important to understand the circuit plasticity mechanisms underlying this process.

How to interpret the action-predictive neurons we found in sensory cortex of trained but not naive animals? We surmise that they may have originally been selective to features present in the target sequence, and then, by virtue of being active at an appropriate time during target presentation, "tagged" during learning as being able to participate in driving the goal-directed response. By a process of associative synaptic potentiation, these neurons might then have become more strongly connected to postsynaptic partners capable of affecting behavior. Results from the auditory modality suggest phenomena consistent with this account: neurons in the auditory cortex sensitive to a frequency range present in a target stimulus eventually become able to drive the GO response.[51,73,104,107] In another whisker-mediated task, in which mice learn to lick in response to a detected whisker deflection, S2-targeting neurons in S1bf also acquire responses predictive of licking.[74]

An alternative is that responses of action-predicting neurons in sensory cortex simply reflect the top-down broadcast of an action choice from higher decision-making centers. If this interpretation is correct, our data have two implications. First, they show that S1bf neurons differ in their sensitivity to the action choice, thus ruling out a homogeneous effect of this broadcast signal. Second, they raise the question of how the sensory information needed to reach the decision is relayed to higher areas. Unravelling this will be important for understanding the specific pathways converting sensory input into a sensory-guided decision.[52]

Experimental Considerations

Our findings underscore the importance to interpreting neuronal responses of measuring behavioral output as well as sensory input, and of including error trials in the analysis. Conversely, our GO/NOGO design does not allow dissociation between the decision to act and the specific choice of action, and thus does not allow us to distinguish between action-predictive and choice-predictive activity.[100,108]

In our findings, learning superimposed behavioral associations onto the responses of neuronal populations in a sensory area. It is likely that the representations of task parameters uncovered here reflect the specific nature of the animals' training: specifically, the fact that mice learned to explicitly discriminate between particular sensory sequences. When an animal is exposed to repeated sequential sensory patterns but not conditioned on them, i.e., is not asked to learn an explicit relationship between the patterns and a goal-directed motor action, changes occurring in primary sensory cortex may be different and include refinements in sensory tuning, leading neurons to enhance categorization[109] and potentially become sensitive to sequence structure over extended periods.[110–113]

STAR★METHODS

Detailed methods are provided in the online version of this paper and include the following:

- KEY RESOURCES TABLE
- RESOURCE AVAILABILITY
  - Lead Contact
  - Materials Availability
  - Data and Code Availability
- EXPERIMENTAL MODEL AND SUBJECT DETAILS
- METHOD DETAILS
  - Surgical procedures
  - Housing and training
  - Optogenetics
  - Two-photon imaging
- QUANTIFICATION AND STATISTICAL ANALYSIS
  - Behavioral analysis
  - Neuronal response analysis

SUPPLEMENTAL INFORMATION

Supplemental Information can be found online at https://doi.org/10.1016/j.cub.2020.10.059.

ACKNOWLEDGMENTS

This work was supported by the UK Medical Research Council (grant number MR/P006639/1) and the University of Sussex Internal Research Development Fund. We thank V. Jayaraman, R. Kerr, D. Kim, L. Looger, K. Svoboda, and the HHMI Janelia Farm GENIE project for mice expressing the genetically encoded calcium indicator GCaMP6f; Simon Peron for help with the head bar adaptation and cranial window construction; Matteo Carandini and Charu Reddy for advice on the clear skull-cap preparation; Chen Qian for help with the optogenetics set-up; Moira Eley for help with animal maintenance and training; and members of the Maravall lab for comments on the manuscript.

AUTHOR CONTRIBUTIONS

M.R.B. and M.B. designed and conducted experiments and analyzed data. E.G. conducted experiments. P.K. analyzed data. M.M. conceived the project, designed experiments, analyzed data, and wrote the manuscript.

DECLARATION OF INTERESTS

M.R.B. is now employed by Scientifica, manufacturer of the two-photon microscope used in the study.

Received: April 17, 2020
Revised: September 1, 2020
Accepted: October 20, 2020
Published: November 12, 2020

REFERENCES

1. Phillips, J.R., Johansson, R.S., and Johnson, K.O. (1990). Representation of braille characters in human nerve fibres. Exp. Brain Res. *81*, 589–592.
2. Phillips, J.R., Johansson, R.S., and Johnson, K.O. (1992). Responses of human mechanoreceptive afferents to embossed dot arrays scanned across fingerpad skin. J. Neurosci. *12*, 827–839.
3. Johnson, K.O. (2001). The roles and functions of cutaneous mechanoreceptors. Curr. Opin. Neurobiol. *11*, 455–461.
4. Arabzadeh, E., Panzeri, S., and Diamond, M.E. (2006). Deciphering the spike train of a sensory neuron: counts and temporal patterns in the rat whisker pathway. J. Neurosci. *26*, 9216–9226.
5. Montemurro, M.A., Panzeri, S., Maravall, M., Alenda, A., Bale, M.R., Brambilla, M., and Petersen, R.S. (2007). Role of precise spike timing in coding of dynamic vibrissa stimuli in somatosensory thalamus. J. Neurophysiol. *98*, 1871–1882.
6. Mackevicius, E.L., Best, M.D., Saal, H.P., and Bensmaia, S.J. (2012). Millisecond precision spike timing shapes tactile perception. J. Neurosci. *32*, 15309–15317.
7. Vázquez, Y., Salinas, E., and Romo, R. (2013). Transformation of the neural code for tactile detection from thalamus to cortex. Proc. Natl. Acad. Sci. USA *110*, E2635–E2644.
8. Weber, A.I., Saal, H.P., Lieber, J.D., Cheng, J.W., Manfredi, L.R., Dammann, J.F., 3rd, and Bensmaia, S.J. (2013). Spatial and temporal codes mediate the tactile perception of natural textures. Proc. Natl. Acad. Sci. USA *110*, 17107–17112.
9. Bale, M.R., Campagner, D., Erskine, A., and Petersen, R.S. (2015). Microsecond-scale timing precision in rodent trigeminal primary afferents. J. Neurosci. *35*, 5935–5940.
10. Bale, M.R., Ince, R.A., Santagata, G., and Petersen, R.S. (2015). Efficient population coding of naturalistic whisker motion in the ventro-posterior medial thalamus based on precise spike timing. Front. Neural Circuits *9*, 50.
11. Segundo, J.P., Moore, G.P., Stensaas, L.J., and Bullock, T.H. (1963). Sensitivity of Neurones in Aplysia to Temporal Pattern of Arriving Impulses. J. Exp. Biol. *40*, 643–667.

CellPress
OPEN ACCESS

12. Branco, T., Clark, B.A., and Häusser, M. (2010). Dendritic discrimination of temporal input sequences in cortical neurons. Science *329*, 1671–1675.
13. Hyde, R.A., and Strowbridge, B.W. (2012). Mnemonic representations of transient stimuli and temporal sequences in the rodent hippocampus in vitro. Nat. Neurosci. *15*, 1430–1438.
14. Ju, H., Dranias, M.R., Banumurthy, G., and VanDongen, A.M. (2015). Spatiotemporal memory is an intrinsic property of networks of dissociated cortical neurons. J. Neurosci. *35*, 4040–4051.
15. Díaz-Quesada, M., Martini, F.J., Ferrati, G., Bureau, I., and Maravall, M. (2014). Diverse thalamocortical short-term plasticity elicited by ongoing stimulation. J. Neurosci. *34*, 515–526.
16. Bale, M.R., Bitzidou, M., Pitas, A., Brebner, L.S., Khazim, L., Anagnou, S.T., Stevenson, C.D., and Maravall, M. (2017). Learning and recognition of tactile temporal sequences by mice and humans. eLife *6*, https://doi.org/10.7554/eLife.27333.
17. Petersen, C.C.H. (2019). Sensorimotor processing in the rodent barrel cortex. Nat. Rev. Neurosci. *20*, 533–546.
18. Maravall, M., Petersen, R.S., Fairhall, A.L., Arabzadeh, E., and Diamond, M.E. (2007). Shifts in coding properties and maintenance of information transmission during adaptation in barrel cortex. PLoS Biol. *5*, e19.
19. Stüttgen, M.C., and Schwarz, C. (2010). Integration of vibrotactile signals for whisker-related perception in rats is governed by short time constants: comparison of neurometric and psychometric detection performance. J. Neurosci. *30*, 2060–2069.
20. Estebanez, L., El Boustani, S., Destexhe, A., and Shulz, D.E. (2012). Correlated input reveals coexisting coding schemes in a sensory cortex. Nat. Neurosci. *15*, 1691–1699.
21. Waiblinger, C., Brugger, D., and Schwarz, C. (2015). Vibrotactile discrimination in the rat whisker system is based on neuronal coding of instantaneous kinematic cues. Cereb. Cortex *25*, 1093–1106.
22. McGuire, L.M., Telian, G., Laboy-Juárez, K.J., Miyashita, T., Lee, D.J., Smith, K.A., and Feldman, D.E. (2016). Short time-scale sensory coding in S1 during discrimination of whisker vibrotactile sequences. PLoS Biol. *14*, e1002549.
23. Pitas, A., Albarracín, A.L., Molano-Mazón, M., and Maravall, M. (2017). Variable temporal integration of stimulus patterns in the mouse barrel cortex. Cereb. Cortex *27*, 1758–1764.
24. Zuo, Y., and Diamond, M.E. (2019). Texture Identification by Bounded Integration of Sensory Cortical Signals. Curr Biol *29*, 1425–1435.e5.
25. Zhao, S., Ting, J.T., Atallah, H.E., Qiu, L., Tan, J., Gloss, B., Augustine, G.J., Deisseroth, K., Luo, M., Graybiel, A.M., and Feng, G. (2011). Cell type–specific channelrhodopsin-2 transgenic mice for optogenetic dissection of neural circuitry function. Nat. Methods *8*, 745–752.
26. Scott, B.B., Constantinople, C.M., Akrami, A., Hanks, T.D., Brody, C.D., and Tank, D.W. (2017). Fronto-parietal Cortical Circuits Encode Accumulated Evidence with a Diversity of Timescales. Neuron *95*, 385–398.e5.
27. Runyan, C.A., Piasini, E., Panzeri, S., and Harvey, C.D. (2017). Distinct timescales of population coding across cortex. Nature *548*, 92–96.
28. Akrami, A., Kopec, C.D., Diamond, M.E., and Brody, C.D. (2018). Posterior parietal cortex represents sensory history and mediates its effects on behaviour. Nature *554*, 368–372.
29. Fassihi, A., Akrami, A., Pulecchi, F., Schönfelder, V.H., and Diamond, M.E. (2017). Transformation of perception from sensory to motor cortex. Curr Biol *27*, 1585–1596.e6.
30. Zagha, E., Ge, X., and McCormick, D.A. (2015). Competing neural ensembles in motor cortex gate goal-directed motor output. Neuron *88*, 565–577.
31. Hernández, A., Nácher, V., Luna, R., Zainos, A., Lemus, L., Alvarez, M., Vázquez, Y., Camarillo, L., and Romo, R. (2010). Decoding a perceptual decision process across cortex. Neuron *66*, 300–314.
32. Zuo, Y., Safaai, H., Notaro, G., Mazzoni, A., Panzeri, S., and Diamond, M.E. (2015). Complementary contributions of spike timing and spike rate to perceptual decisions in rat S1 and S2 cortex. Curr. Biol. *25*, 357–363.
33. Chen, J.L., Voigt, F.F., Javadzadeh, M., Krueppel, R., and Helmchen, F. (2016). Long-range population dynamics of anatomically defined neocortical networks. eLife *5*, https://doi.org/10.7554/eLife.14679.
34. Kwon, S.E., Yang, H., Minamisawa, G., and O'Connor, D.H. (2016). Sensory and decision-related activity propagate in a cortical feedback loop during touch perception. Nat. Neurosci. *19*, 1243–1249.
35. Yang, H., Kwon, S.E., Severson, K.S., and O'Connor, D.H. (2016). Origins of choice-related activity in mouse somatosensory cortex. Nat. Neurosci. *19*, 127–134.
36. Ebbesen, C.L., and Brecht, M. (2017). Motor cortex - to act or not to act? Nat. Rev. Neurosci. *18*, 694–705.
37. Stoltz, S., Humm, J.L., and Schallert, T. (1999). Cortical injury impairs contralateral forelimb immobility during swimming: a simple test for loss of inhibitory motor control. Behav. Brain Res. *106*, 127–132.
38. Dana, H., Chen, T.W., Hu, A., Shields, B.C., Guo, C., Looger, L.L., Kim, D.S., and Svoboda, K. (2014). Thy1-GCaMP6 transgenic mice for neuronal population imaging in vivo. PLoS ONE *9*, e108697.
39. Peron, S.P., Freeman, J., Iyer, V., Guo, C., and Svoboda, K. (2015). A Cellular Resolution Map of Barrel Cortex Activity during Tactile Behavior. Neuron *86*, 783–799.
40. Campagner, D., Evans, M.H., Loft, M.S.E., and Petersen, R.S. (2018). What the whiskers tell the brain. Neuroscience *368*, 95–108.
41. Bale, M.R., and Maravall, M. (2018). Organization of sensory feature selectivity in the whisker system. Neuroscience *368*, 70–80.
42. Raposo, D., Kaufman, M.T., and Churchland, A.K. (2014). A category-free neural population supports evolving demands during decision-making. Nat. Neurosci. *17*, 1784–1792.
43. O'Connor, D.H., Clack, N.G., Huber, D., Komiyama, T., Myers, E.W., and Svoboda, K. (2010). Vibrissa-based object localization in head-fixed mice. J. Neurosci. *30*, 1947–1967.
44. O'Connor, D.H., Hires, S.A., Guo, Z.V., Li, N., Yu, J., Sun, Q.Q., Huber, D., and Svoboda, K. (2013). Neural coding during active somatosensation revealed using illusory touch. Nat. Neurosci. *16*, 958–965.
45. Miyashita, T., and Feldman, D.E. (2013). Behavioral detection of passive whisker stimuli requires somatosensory cortex. Cereb. Cortex *23*, 1655–1662.
46. Sachidhanandam, S., Sreenivasan, V., Kyriakatos, A., Kremer, Y., and Petersen, C.C. (2013). Membrane potential correlates of sensory perception in mouse barrel cortex. Nat. Neurosci. *16*, 1671–1677.
47. Guo, Z.V., Li, N., Huber, D., Ophir, E., Gutnisky, D., Ting, J.T., Feng, G., and Svoboda, K. (2014). Flow of cortical activity underlying a tactile decision in mice. Neuron *81*, 179–194.
48. Hutson, K.A., and Masterton, R.B. (1986). The sensory contribution of a single vibrissa's cortical barrel. J. Neurophysiol. *56*, 1196–1223.
49. Hong, Y.K., Lacefield, C.O., Rodgers, C.C., and Bruno, R.M. (2018). Sensation, movement and learning in the absence of barrel cortex. Nature *561*, 542–546.
50. Whitfield, I.C. (1979). The object of the sensory cortex. Brain Behav. Evol. *16*, 129–154.
51. Ceballo, S., Piwkowska, Z., Bourg, J., Daret, A., and Bathellier, B. (2019). Targeted cortical manipulation of auditory perception. Neuron *104*, 1168–1179.e5.
52. Romo, R., and de Lafuente, V. (2013). Conversion of sensory signals into perceptual decisions. Prog. Neurobiol. *103*, 41–75.
53. Masquelier, T., and Thorpe, S.J. (2007). Unsupervised learning of visual features through spike timing dependent plasticity. PLoS Comput. Biol. *3*, e31.
54. Lee, T.P., and Buonomano, D.V. (2012). Unsupervised formation of vocalization-sensitive neurons: a cortical model based on short-term and homeostatic plasticity. Neural Comput. *24*, 2579–2603.

55. Cui, Y., Ahmad, S., and Hawkins, J. (2016). Continuous online sequence learning with an unsupervised neural network model. Neural Comput. *28*, 2474–2504.

56. Marr, D. (2010). Vision: A Computational Investigation into the Human Representation and Processing of Visual Information (Cambridge, MA: MIT Press).

57. Keller, G.B., and Mrsic-Flogel, T.D. (2018). Predictive Processing: A Canonical Cortical Computation. Neuron *100*, 424–435.

58. Britten, K.H., Newsome, W.T., Shadlen, M.N., Celebrini, S., and Movshon, J.A. (1996). A relationship between behavioral choice and the visual responses of neurons in macaque MT. Vis. Neurosci. *13*, 87–100.

59. Selezneva, E., Scheich, H., and Brosch, M. (2006). Dual time scales for categorical decision making in auditory cortex. Curr. Biol. *16*, 2428–2433.

60. Shuler, M.G., and Bear, M.F. (2006). Reward timing in the primary visual cortex. Science *311*, 1606–1609.

61. Scheich, H., Brechmann, A., Brosch, M., Budinger, E., and Ohl, F.W. (2007). The cognitive auditory cortex: task-specificity of stimulus representations. Hear. Res. *229*, 213–224.

62. Nienborg, H., and Cumming, B.G. (2009). Decision-related activity in sensory neurons reflects more than a neuron's causal effect. Nature *459*, 89–92.

63. Niell, C.M., and Stryker, M.P. (2010). Modulation of visual responses by behavioral state in mouse visual cortex. Neuron *65*, 472–479.

64. Jaramillo, S., and Zador, A.M. (2011). The auditory cortex mediates the perceptual effects of acoustic temporal expectation. Nat. Neurosci. *14*, 246–251.

65. Keller, G.B., Bonhoeffer, T., and Hübener, M. (2012). Sensorimotor mismatch signals in primary visual cortex of the behaving mouse. Neuron *74*, 809–815.

66. Goltstein, P.M., Coffey, E.B., Roelfsema, P.R., and Pennartz, C.M. (2013). In vivo two-photon Ca2+ imaging reveals selective reward effects on stimulus-specific assemblies in mouse visual cortex. J. Neurosci. *33*, 11540–11555.

67. Saleem, A.B., Ayaz, A., Jeffery, K.J., Harris, K.D., and Carandini, M. (2013). Integration of visual motion and locomotion in mouse visual cortex. Nat. Neurosci. *16*, 1864–1869.

68. Stănişor, L., van der Togt, C., Pennartz, C.M., and Roelfsema, P.R. (2013). A unified selection signal for attention and reward in primary visual cortex. Proc. Natl. Acad. Sci. USA *110*, 9136–9141.

69. Atiani, S., David, S.V., Elgueda, D., Locastro, M., Radtke-Schuller, S., Shamma, S.A., and Fritz, J.B. (2014). Emergent selectivity for task-relevant stimuli in higher-order auditory cortex. Neuron *82*, 486–499.

70. Schneider, D.M., Nelson, A., and Mooney, R. (2014). A synaptic and circuit basis for corollary discharge in the auditory cortex. Nature *513*, 189–194.

71. Kato, H.K., Gillet, S.N., and Isaacson, J.S. (2015). Flexible Sensory Representations in Auditory Cortex Driven by Behavioral Relevance. Neuron *88*, 1027–1039.

72. Poort, J., Khan, A.G., Pachitariu, M., Nemri, A., Orsolic, I., Krupic, J., Bauza, M., Sahani, M., Keller, G.B., Mrsic-Flogel, T.D., and Hofer, S.B. (2015). Learning Enhances Sensory and Multiple Non-sensory Representations in Primary Visual Cortex. Neuron *86*, 1478–1490.

73. Tsunada, J., Liu, A.S., Gold, J.I., and Cohen, Y.E. (2016). Causal contribution of primate auditory cortex to auditory perceptual decision-making. Nat. Neurosci. *19*, 135–142.

74. Yamashita, T., and Petersen, C.Ch. (2016). Target-specific membrane potential dynamics of neocortical projection neurons during goal-directed behavior. eLife *5*, https://doi.org/10.7554/eLife.15798.

75. Leinweber, M., Ward, D.R., Sobczak, J.M., Attinger, A., and Keller, G.B. (2017). A Sensorimotor Circuit in Mouse Cortex for Visual Flow Predictions. Neuron *95*, 1420–1432.e5.

76. Bagur, S., Averseng, M., Elgueda, D., David, S., Fritz, J., Yin, P., Shamma, S., Boubenec, Y., and Ostojic, S. (2018). Go/No-Go task engagement enhances population representation of target stimuli in primary auditory cortex. Nat. Commun. *9*, 2529.

77. Francis, N.A., Winkowski, D.E., Sheikhattar, A., Armengol, K., Babadi, B., and Kanold, P.O. (2018). Small Networks Encode Decision-Making in Primary Auditory Cortex. Neuron *97*, 885–897.e6.

78. Pakan, J.M.P., Currie, S.P., Fischer, L., and Rochefort, N.L. (2018). The Impact of Visual Cues, Reward, and Motor Feedback on the Representation of Behaviorally Relevant Spatial Locations in Primary Visual Cortex. Cell Rep. *24*, 2521–2528.

79. Ramesh, R.N., Burgess, C.R., Sugden, A.U., Gyetvan, M., and Andermann, M.L. (2018). Intermingled Ensembles in Visual Association Cortex Encode Stimulus Identity or Predicted Outcome. Neuron *100*, 900–915.e9.

80. Chéreau, R., Bawa, T., Fodoulian, L., Carleton, A., Pagès, S., and Holtmaat, A. (2020). Dynamic perceptual feature selectivity in primary somatosensory cortex upon reversal learning. Nat. Commun. *11*, https://doi.org/10.1038/s41467-020-17005-x.

81. Elgueda, D., Duque, D., Radtke-Schuller, S., Yin, P., David, S.V., Shamma, S.A., and Fritz, J.B. (2019). State-dependent encoding of sound and behavioral meaning in a tertiary region of the ferret auditory cortex. Nat. Neurosci. *22*, 447–459.

82. Guo, L., Weems, J.T., Walker, W.I., Levichev, A., and Jaramillo, S. (2019). Choice-Selective Neurons in the Auditory Cortex and in Its Striatal Target Encode Reward Expectation. J. Neurosci. *39*, 3687–3697.

83. Musall, S., Kaufman, M.T., Juavinett, A.L., Gluf, S., and Churchland, A.K. (2019). Single-trial neural dynamics are dominated by richly varied movements. Nat. Neurosci. *22*, 1677–1686.

84. Condylis, C., Lowet, E., Ni, J., Bistrong, K., Ouellette, T., Josephs, N., and Chen, J.L. (2020). Context-Dependent Sensory Processing across Primary and Secondary Somatosensory Cortex. Neuron *106*, 515–525.e5.

85. Henschke, J.U., Dylda, E., Katsanevaki, D., Dupuy, N., Currie, S.P., Amvrosiadis, T., Pakan, J.M.P., and Rochefort, N.L. (2020). Reward Association Enhances Stimulus-Specific Representations in Primary Visual Cortex. Curr. Biol. *30*, 1866–1880.e5.

86. Koay, S.A., Thiberge, S.Y., Brody, C.D., and Tank, D.W. (2019). Neural Correlates of Cognition in Primary Visual versus Neighboring Posterior Cortices during Visual Evidence-Accumulation-based Navigation. bioRxiv. https://doi.org/10.1101/782847.

87. Lacefield, C.O., Pnevmatikakis, E.A., Paninski, L., and Bruno, R.M. (2019). Reinforcement Learning Recruits Somata and Apical Dendrites across Layers of Primary Sensory Cortex. Cell Rep *26*, 2000–2008.e2.

88. Machens, C.K., Romo, R., and Brody, C.D. (2010). Functional, but not anatomical, separation of "what" and "when" in prefrontal cortex. J. Neurosci. *30*, 350–360.

89. Hyman, J.M., Whitman, J., Emberly, E., Woodward, T.S., and Seamans, J.K. (2013). Action and outcome activity state patterns in the anterior cingulate cortex. Cereb. Cortex *23*, 1257–1268.

90. Mante, V., Sussillo, D., Shenoy, K.V., and Newsome, W.T. (2013). Context-dependent computation by recurrent dynamics in prefrontal cortex. Nature *503*, 78–84.

91. Rigotti, M., Barak, O., Warden, M.R., Wang, X.J., Daw, N.D., Miller, E.K., and Fusi, S. (2013). The importance of mixed selectivity in complex cognitive tasks. Nature *497*, 585–590.

92. Pinto, L., and Dan, Y. (2015). Cell-Type-Specific Activity in Prefrontal Cortex during Goal-Directed Behavior. Neuron *87*, 437–450.

93. Park, I.M., Meister, M.L., Huk, A.C., and Pillow, J.W. (2014). Encoding and decoding in parietal cortex during sensorimotor decision-making. Nat. Neurosci. *17*, 1395–1403.

94. Zingg, B., Hintiryan, H., Gou, L., Song, M.Y., Bay, M., Bienkowski, M.S., Foster, N.N., Yamashita, S., Bowman, I., Toga, A.W., and Dong, H.W. (2014). Neural networks of the mouse neocortex. Cell *156*, 1096–1111.

CellPress
OPEN ACCESS

95. Zhang, S., Xu, M., Chang, W.C., Ma, C., Hoang Do, J.P., Jeong, D., Lei, T., Fan, J.L., and Dan, Y. (2016). Organization of long-range inputs and outputs of frontal cortex for top-down control. Nat. Neurosci. *19*, 1733–1742.

96. Allen, W.E., Kauvar, I.V., Chen, M.Z., Richman, E.B., Yang, S.J., Chan, K., Gradinaru, V., Deverman, B.E., Luo, L., and Deisseroth, K. (2017). Global Representations of Goal-Directed Behavior in Distinct Cell Types of Mouse Neocortex. Neuron *94*, 891–907.e6.

97. Makino, H., Ren, C., Liu, H., Kim, A.N., Kondapaneni, N., Liu, X., Kuzum, D., and Komiyama, T. (2017). Transformation of Cortex-wide Emergent Properties during Motor Learning. Neuron *94*, 880–890.e8.

98. Orsolic, I., Rio, M., Mrsic-Flogel, T.D., and Znamenskiy, P. (2019). Mesoscale cortical dynamics reflect the interaction of sensory evidence and temporal expectation during perceptual decision-making. bioRxiv. https://doi.org/10.1101/552026.

99. Stringer, C., Pachitariu, M., Steinmetz, N., Reddy, C.B., Carandini, M., and Harris, K.D. (2019). Spontaneous behaviors drive multidimensional, brainwide activity. Science *364*, 255.

100. Steinmetz, N.A., Zatka-Haas, P., Carandini, M., and Harris, K.D. (2019). Distributed coding of choice, action and engagement across the mouse brain. Nature *576*, 266–273.

101. Crapse, T.B., and Sommer, M.A. (2008). Corollary discharge across the animal kingdom. Nat. Rev. Neurosci. *9*, 587–600.

102. Siegel, M., Buschman, T.J., and Miller, E.K. (2015). Cortical information flow during flexible sensorimotor decisions. Science *348*, 1352–1355.

103. de Lafuente, V., and Romo, R. (2006). Neural correlate of subjective sensory experience gradually builds up across cortical areas. Proc. Natl. Acad. Sci. USA *103*, 14266–14271.

104. Znamenskiy, P., and Zador, A.M. (2013). Corticostriatal neurons in auditory cortex drive decisions during auditory discrimination. Nature *497*, 482–485.

105. Yartsev, M.M., Hanks, T.D., Yoon, A.M., and Brody, C.D. (2018). Causal contribution and dynamical encoding in the striatum during evidence accumulation. eLife *7*, https://doi.org/10.7554/eLife.34929.

106. Le Merre, P., Esmaeili, V., Charriere, E., Galan, K., Salin, P.A., Petersen, C.C.H., and Crochet, S. (2018). Reward-Based Learning Drives Rapid Sensory Signals in Medial Prefrontal Cortex and Dorsal Hippocampus Necessary for Goal-Directed Behavior. Neuron *97*, 83–91.e5.

107. Xiong, Q., Znamenskiy, P., and Zador, A.M. (2015). Selective corticostriatal plasticity during acquisition of an auditory discrimination task. Nature *521*, 348–351.

108. Carandini, M., and Churchland, A.K. (2013). Probing perceptual decisions in rodents. Nat. Neurosci. *16*, 824–831.

109. Xin, Y., Zhong, L., Zhang, Y., Zhou, T., Pan, J., and Xu, N.-l. (2019). Sensory-to-Category Transformation via Dynamic Reorganization of Ensemble Structures in Mouse Auditory Cortex. Neuron *103*, 909–921.e6.

110. Xu, S., Jiang, W., Poo, M.M., and Dan, Y. (2012). Activity recall in a visual cortical ensemble. Nat Neurosci *15*, 449–455.

111. Gavornik, J.P., and Bear, M.F. (2014). Learned spatiotemporal sequence recognition and prediction in primary visual cortex. Nat. Neurosci. *17*, 732–737.

112. Homann, J., Koay, S.A., Glidden, A.M., Tank, D.W., and Berry, M.J. (2017). Predictive coding of novel versus familiar stimuli in the primary visual cortex. biorxiv. https://doi.org/10.1101/197608.

113. Libby, A., and Buschman, T.J. (2019). Rotational Dynamics Reduce Interference Between Sensory and Memory Representations. bioRxiv. https://doi.org/10.1101/641159.

114. Schindelin, J., Arganda-Carreras, I., Frise, E., Kaynig, V., Longair, M., Pietzsch, T., Preibisch, S., Rueden, C., Saalfeld, S., Schmid, B., et al. (2012). Fiji: an open-source platform for biological-image analysis. Nat. Methods *9*, 676–682.

115. Pachitariu, M., Stringer, C., Dipoppa, M., Schröder, S., Rossi, L.F., Dalgleish, H., Carandini, M., and Harris, K.D. (2017). Suite2p: beyond 10,000 neurons with standard two-photon microscopy. bioRxiv. https://doi.org/10.1101/061507.

116. Pedregosa, F., Varoquaux, G., Gramfort, A., Michel, V., Thirion, B., Grisel, O., Blondel, M., Prettenhofer, P., Weiss, R., Dubourg, V., et al. (2011). Scikit-learn: Machine Learning in Python. J. Mach. Learn. Res. *12*, 2825–2830.

117. Guo, Z.V., Hires, S.A., Li, N., O'Connor, D.H., Komiyama, T., Ophir, E., Huber, D., Bonardi, C., Morandell, K., Gutnisky, D., et al. (2014). Procedures for behavioral experiments in head-fixed mice. PLoS ONE *9*, e88678.

118. Tucci, V., Hardy, A., and Nolan, P.M. (2006). A comparison of physiological and behavioural parameters in C57BL/6J mice undergoing food or water restriction regimes. Behav. Brain Res. *173*, 22–29.

119. Sanders, J.I., and Kepecs, A. (2014). A low-cost programmable pulse generator for physiology and behavior. Front. Neuroeng. *7*, 43.

120. Burgess, C.P., Lak, A., Steinmetz, N.A., Zatka-Haas, P., Bai Reddy, C., Jacobs, E.A.K., Linden, J.F., Paton, J.J., Ranson, A., Schröder, S., et al. (2017). High-Yield Methods for Accurate Two-Alternative Visual Psychophysics in Head-Fixed Mice. Cell Rep. *20*, 2513–2524.

121. Goard, M.J., Pho, G.N., Woodson, J., and Sur, M. (2016). Distinct roles of visual, parietal, and frontal motor cortices in memory-guided sensorimotor decisions. eLife *5*, e13764.

122. Chen, T.W., Wardill, T.J., Sun, Y., Pulver, S.R., Renninger, S.L., Baohan, A., Schreiter, E.R., Kerr, R.A., Orger, M.B., Jayaraman, V., et al. (2013). Ultrasensitive fluorescent proteins for imaging neuronal activity. Nature *499*, 295–300.

123. Kerlin, A.M., Andermann, M.L., Berezovskii, V.K., and Reid, R.C. (2010). Broadly tuned response properties of diverse inhibitory neuron subtypes in mouse visual cortex. Neuron *67*, 858–871.

124. Evans, M., Petersen, R.D., and Humphries, M.D. (2019). On the use of calcium deconvolution algorithms in practical contexts. bioRxiv. https://doi.org/10.1101/871137.

125. Wei, Z., Lin, B.-J., Chen, T.-W., Daie, K., Svoboda, K., and Druckmann, S. (2020). A comparison of neuronal population dynamics measured with calcium imaging and electrophysiology. PLoS Comput. Biol. *16*, e1008198, https://doi.org/10.1371/journal.pcbi.1008198.

126. Sabatini, B.L. (2019). The impact of reporter kinetics on the interpretation of data gathered with fluorescent reporters. bioRxiv. https://doi.org/10.1101/834895.

STAR★METHODS

KEY RESOURCES TABLE

REAGENT or RESOURCE	SOURCE	IDENTIFIER
Deposited Data		
Dataset and specialized code	This study	https://doi.org/10.25377/sussex.12573881
Experimental Models: Organisms/Strains		
C57BL/6J mice	The Jackson Laboratory	JAX:000664
VGAT-ChR2-EYFP mice	The Jackson Laboratory	JAX:014548
Thy1-GCaMP6f mice, founder line 5.5	The Jackson Laboratory	JAX:024276
Thy1-GCaMP6f mice, founder line 5.17	The Jackson Laboratory	JAX:025393
Software and Algorithms		
MATLAB	Mathworks	RRID:SCR_001622; http://www.mathworks.com/products/matlab/
Python	Python Software Foundation	RRID:SCR_008394; https://www.python.org
Fiji	[114]	RRID:SCR_002285; https://fiji.sc/
Bpod	Sanworks LLC	RRID:SCR_015943; https://github.com/sanworks
Scanimage	Vidrio Technologies	RRID:SCR_014307; http://scanimage.vidriotechnologies.com/
Suite2p	[115]	RRID:SCR_016434; http://www.suite2p.org/
R	The R Foundation	RRID:SCR_001905; https://www.r-project.org
Sklearn	[116]	RRID:SCR_019053; https://scikit-learn.org/stable/modules/generated/sklearn.decomposition.NMF.html

RESOURCE AVAILABILITY

Lead Contact

Further information and requests for resources and reagents should be directed to and will be fulfilled by the Lead Contact, Miguel Maravall (m.maravall@sussex.ac.uk)

Materials Availability

This study did not generate unique new reagents.

Data and Code Availability

The datasets generated during this study are available at Figshare under DOI https://doi.org/10.25377/sussex.12573881.

EXPERIMENTAL MODEL AND SUBJECT DETAILS

All procedures were conducted in accordance with national (UK Animals (Scientific Procedures) Act 1986) and international (European Union directive 2010/63/EU) regulations for the care and use of animals in research, and under the authority of Project License 70/8400. Personal and project licenses to carry out the work were approved upon institutional (University of Sussex Animal Welfare and Ethical Review Body) and Home Office review. Experimental mice were males on a C57BL/6J background, 4-9 weeks old at the time of initial surgery and bred at the University of Sussex. Animals were group housed until surgery and randomly assigned to experimental groups.

METHOD DETAILS

Surgical procedures

Details of head bar implantation surgery have been published elsewhere.[16,117] Briefly, under aseptic conditions, mice were anaesthetised using 1.5%–2.5% isoflurane in O_2 and placed into a stereotaxic apparatus (Narishige, Japan) with ear bars previously coated with EMLA cream. We monitored anesthetic depth by checking spinal reflexes and breathing rates. Body temperature was maintained at 37°C using a homeothermic heating pad (FHC). Eyes were treated with ophthalmic gel (Viscotears Liquid Gel, Novartis, Switzerland) and the entire scalp washed with povidone-iodine solution. An area of skin was removed (an oval of approximately 15 mm x 10 mm in the sagittal plane) such that all skull landmarks were visible and sufficient skull was accessible to securely fix a titanium or stainless steel head bar. The exposed periosteum was removed and the bone washed using saline solution, dried

with sterile swabs and then scraped with a scalpel blade to aid bonding of glue. Cyanoacrylate glue (Vetbond, 3M) was applied to bind skin edges to the skull and as a thin layer across the exposed skull to aid bonding to the dental acrylic. A custom titanium or stainless steel head bar (dimensions 22.3 × 3.2 × 1.3 mm; design by Karel Svoboda lab, Janelia Farm Research Campus, Howard Hughes Medical Institute, http://bit.ly/jMouseHeadplateHolder)[117] was placed directly onto the wet glue centered just posterior to lambda. Once dry, we scraped the glue surface to improve bonding and fixed the head bar firmly in place by applying dental acrylic (Ortho-Jet, Lang Dental) to the head bar (on top and behind) and the skull (anterior). Mice were given buprenorphine (0.1 mg/kg, I.P.) and further EMLA cream to the paws and ears. Once the acrylic was set, anesthesia was turned off and animals returned to the cage. On the day of surgery and for the next two consecutive days 200 μL of non-steroidal anti-inflammatory drug (Metacam oral suspension 0.5mg/mL; Boehringer Ingelheim) was mixed with food pellets soaked in water until they became mash. Animals were housed individually and allowed to recover for one week post-surgery, with health and weight monitored daily.

Housing and training

Animals were housed in cages with bedding, tubes, running wheels and a plastic plate attached to a custom platform used to provide daily water, and kept on a reverse 50:50 light-dark (LD) cycle.

Water control

To motivate mice to perform the task we employed a water restriction protocol[117] and made water available as a reward for correct discrimination of GO stimuli. Mice cope well physiologically with water restriction, as they are adapted to life in semiarid environments.[118] Dry food was available at all times. We observed a mild increase in motivation when mice were given sunflower seeds before tasks.

Mouse water intake was regulated so that animals were motivated to perform for 200 or more trials per session under our conditions (45%–55% humidity, 20°C and atmospheric pressure; reverse 50:50 LD cycle), while remaining active and healthy. The water control protocol started 7 days after head bar implantation surgery. A single training session was performed on each day when training was carried out. Animals received water during the training session; reward water intake was determined by weighing the animal before and after the session together with collected faeces, and was typically 0.1-0.4 ml. Mice were then given further *ad libitum* water during a finite (usually 1 min) free drinking period after the end of the session. On days with no training, mice were given free access to 1.5 ml, which corresponds to 50% of average *ad libitum* water intake for C57BL/6J mice (Mouse Phenome Database from the Jackson Laboratory: http://www.jax.org/phenome). The health of animals under water restriction was assessed daily (dehydration, weight, grooming, movement) and a checklist filled. Mice initially lost weight but then increased body mass gradually over the course of water restriction. Sensory discrimination training began after 9 days on water control.

Animal handling and training set-up

Mice were trained to enter a head fixation device using a shaping procedure. We initiated water control one week after head bar implantation. On days 1 and 2 animals were given 1.5 mL of water placed in their cage. On days 3 and 4 animals were introduced to the experimenter. They were first left to smell and explore the experimenter's hand while in their cage, then gently picked up using a tube and returned to the cage several times while given sunflower seeds and water from a syringe. On days 5 and 6 mice were introduced to the head fixation device. They received sunflower seeds and water via a syringe only when inside the device (but not head-fixed). At this stage, mice were grooming and eating in the head fixation apparatus without any signs of distress. On days 7 and 8 animals were given a sunflower seed and after ingestion were head-fixed and given water via a syringe. Animals became accustomed to head fixation and expected to receive water from the spout situated in front of their head. On day 9, mice began the task. Animals were trained in the dark; illumination, if necessary, was provided by a red lamp.

We used two device designs. One design consisted of an acrylic tube (32 mm internal diameter) with its head end cut to enable access to the implanted head bars. The tube was placed on Parafilm or a rubber glove and clamped into a v-shape groove. This support acted to stabilize the tube, collect faeces and prevent mice from grasping stimulus apparatus and the lickport. The second design consisted of a custom 3D-printed treadmill on which mice could locomote freely (design by Leopoldo Petreanu, Champalimaud Centre for the Unknown). A metallic mesh was fixed over the treadmill to surround the mouse's body, allowing the animal to feel comfortably enclosed rather than exposed. The ends of the head bars were inserted into notches on two head fixation clamps and tightened using thumbscrews.

Water rewards were provided through an electrical lickport ending in a spout made from a blunted gauge 13 syringe needle. Water flow from an elevated container was controlled via a solenoid valve (LDHA1233215H, The Lee Company, France). The acrylic tube was lined with aluminum foil. Terminals from an A/D input of a signal processor were connected to the water spout and the foil or the metallic head bar holder, so that tongue contacts with the lick port created brief elevations in voltage consistent with lick durations. This opened the solenoid valve for an adjustable amount of time, delivering 1-2 μL of water. Correct positioning of the lickport was an important aspect of training: in the first sessions it was placed relatively close to the mouth of the animal, to facilitate initial successful collection of rewards, but was gradually moved away from the mouth during training to avoid development of impulsive licking.

Stimulus design and delivery

Stimulus sequences were constructed in MATLAB (Mathworks, USA). Stimulus playback and trial control was performed either via a signal processor (RP2.1, TDT, USA) controlled with ActiveX, or via a Bpod/PulsePal (Sanworks LLC) open-source Arduino-based system[119] controlled with MATLAB. Trial outcomes were recorded in MATLAB. Trials began with a 'stimulus presentation period' lasting 550 ms, in which the sequence was delivered. Mice were not rewarded or punished for licking during this period. At the

end of this stimulation period followed a 'response period' (1.5 s) where mice needed either to lick or refrain from licking, depending on stimulus sequence.

Multiple whiskers on one side of the snout were trimmed to 1 cm and placed into a 10 mm^2 metallic mesh grid (at least 3 whiskers in the grid), glued to a piezoelectric actuator (PL127.11, Physik Instrumente, Germany) and positioned ~1 mm from the animal's fur.

To ensure that mice detected the lowest amplitude filtered noise part of the sequence stimuli (Figure 1B), animals (n = 3) that had successfully learned normal detection were trained to detect the lowest amplitude syllable. All of the animals accomplished high performance on detection within a single session (mean 81% correct, SD 7.83%; n = 4 sessions).

Optogenetics

To suppress activity in dorsal cortical areas, we photostimulated channelrhodopsin-2 (ChR2) in GABAergic interneurons of VGAT-ChR2-EYFP mice[25] (breeding pairs from The Jackson Laboratory; stock number: 014548). Three mice were used to measure light transmission through the clear-skull cap preparation, one mouse to verify expression of ChR2, and six mice to characterize photo-inhibition. Of these, four were used for behavioral training and optogenetics experiments.

Surgeries

Mice were implanted with a head bar as described above. At the same time, a clear-skull cap was added,[47,100,120] as follows. After marking bregma using a surgical marker, covering the bone surface with a thin layer of cyanoacrylate glue and allowing the glue to dry, two to three thin layers of UV curing optical adhesive (Norland Optical Adhesives #81, Norland Products Inc.) were applied to the skull and cured using a UV LED (DC4100, Thorlabs). Next, the headbar was attached to the skull and fixed with dental cement. To avoid scratches during the animal's recovery and training period and keep the clear-skull cap's surface smooth, it was covered with a silicon sealant (Kwik-Cast, World Precision Instruments). The sealant was removed prior to each experiment and a new layer applied before the animal returned to its cage.

Photoinhibition

Light from a 473 nm modulated diode laser system (Cobolt 06-MLD, Laserlines) was controlled with digital modulation (< 2.5 ns rise time). The laser head was fiber coupled (FC/PC) to a 2 m length multi-mode optical fiber (200 μm diameter, Laserlines). Light coming out of the fiber was collimated using an adjustable collimator (350-700 nm, CFC-8X-A, Thorlabs) and passed through a coated plano convex lens (LA1951-A, Thorlabs), to be focused onto the surface of the clear-skull cap (Figure S2A). Light modulation followed a 50 Hz square wave control signal generated by a voltage pulse generator (PulsePal, Sanworks). Laser power was calibrated using a handheld power meter (NT54-018, Edmund Optics). The laser beam had a Gaussian profile (Figure S2B; FWHM 364 μm), determined using a CMOS camera (DCC1545, Thorlabs; pixel size 5.2 μm) and analyzed with Fiji.

Light transmission through the clear-skull cap was measured on a separate group of mice that underwent the preparation surgery and were then euthanized (n = 3 mice). The clear-skull cap (skull, cyanoacrylate glue and UV curing optical adhesive) was next isolated and laser power measured before and after passing through it. Light transmission was 36 ± 2% (SD). After calibration, laser power was set to approximately 3.4 mW at the brain surface. We estimated the spatial spread of optogenetic activity suppression in VGAT-ChR2-EYFP mice by carrying out immunohistological labeling of activity-dependent cFos expression after we had stimulated interneuron activity by illuminating with the blue laser (Figure S2C).[121]

At the beginning of each experiment, the mouse was head-fixed and silicon sealant removed. The laser beam was positioned over bregma and subsequently moved to the brain area of interest with a motorized manipulator (MP-225, Sutter Instruments). A single area was perturbed in each session.

Coordinates used for optogenetic suppression were: for S1bf, 1 mm anteroposterior from bregma (AP), 3 mm mediolateral (ML); S2, 1.2 mm AP, 4.2 mm ML; PPC, 2 mm AP, 1.7 mm ML; and wM1, 1.1 mm anterior to bregma, 0.9 mm ML. Distances between optogenetic stimulation sites centered over S1bf, S2, PPC and wM1 were at least 1.2 mm, > 3x the FWHM of the laser beam. The absence of a systematic impact of optogenetic PPC suppression suggests that the results observed when the laser was centered over S1bf/S2 cannot be attributed to a generic effect on cortical activity. Moreover, our stimulation sites in PPC and S1bf, which yielded contrasting effects (Figures 2B and 2E), were closer together than those in S1bf and S2, which yielded similar behavior (Figures 2B and 2C). Some bleeding through of S2-centered laser light into S1bf cannot be fully ruled out, although the similar impact on task performance of optogenetic suppression centered over S2 and S1bf suggests an equally direct effect of suppression at both sites.

Two-photon imaging

Surgeries

Thy1-GCaMP6 mice expressing GCaMP6f in pyramidal neurons[38,122] (founder lines GP5.5 and 5.17) were implanted with a head bar as described above. A circular 3 mm diameter craniotomy was made to expose the brain. A cranial window, consisting of a 3 mm circular coverslip and a 5 mm circular coverslip (Harvard Instruments), was placed over the craniotomy and secured in place with cyanoacrylate tissue sealant (Vetbond, 3M). Following recovery, mice were trained to perform the task while head-fixed under the two-photon microscope, using a shaping procedure as described above. On concluding the experiments, we checked for specificity of GCaMP6f expression in excitatory neurons by staining Thy1-GCaMP6 mice for VGAT expression (mean 1.4% of Thy1-positive neurons expressed VGAT, range 1.0%–1.9%; n = 4 mice).

Imaging

A two-photon microscope with galvanometric scanning (Scientifica) was controlled by Scanimage software (Vidrio Technologies). Illumination was provided by a Ti:sapphire Chameleon Vision S laser (Coherent Technologies) tuned to 940 nm and focused through a 20x/1.0NA water immersion objective (Olympus). Laser power under the objective was 100-120 mW. Frame scanning (256x100 pixels) was performed at 10.8 Hz. Because the duration of trial epochs and of different segments in the stimulus sequences was well over 100 ms, our imaging temporal resolution was enough to permit detection of neurons sensitive to different epochs or sequence segments (e.g., Figure 5A).

Image processing

Raw images were de-interleaved in Fiji[114] to extract the stimulus synchronization channel from the images. Image processing was then carried out using Suite2p[115] running in MATLAB. After registration and motion correction, ROIs were automatically detected and manually adjusted. Raw fluorescence was extracted for each ROI and corrected for neuropil contamination ($F = F_{raw} - \alpha F_{neuropil}$).[122,123] Baseline fluorescence F_0 was computed using a 2-3 min sliding window, using the 5th percentile of the raw distribution within the window for highly skewed cells, or the median for cells with a symmetric distribution.[39]

QUANTIFICATION AND STATISTICAL ANALYSIS

Details on choice of tests, values and meaning of n, and statistical measures, are given next to the corresponding result. No statistical methods were used to predetermine sample size.

Behavioral analysis

Analyses were conducted in MATLAB and R. We quantified behavioral performance as in,[16] using the percent correct metric (hits + CRs)/(number of trials in sliding window) determined over a 50-trial sliding window during the course of a session and corrected for the proportions of GO and NOGO trials. To obtain an upper bound on average decision times in a session, we determined when, on average, the lick rates for GO and NOGO trials began to diverge during the course of a trial (discriminative lick latency, DLL).[16] We first subtracted the lick rate curve for NOGO trials from that for GO trials, and set a threshold for when this subtracted curve became positive (i.e., when GO licks surpassed NOGO licks) by determining the 95% confidence limit for the distribution of subtracted lick rate curves throughout the trial for 50 randomly subsampled sets of GO and NOGO trials (time points sampled at 100 ms resolution). We determined the DLL both including and excluding 'acausal' licks, i.e., those generated by the animal before it was possible to distinguish whether the trial was GO or NOGO; this made no difference to the conclusions of the analysis.

Rather than reflecting deliberation, the covariation between DLL and performance could be a consequence of well-trained animals reaching an understanding that early licking brings no reward. In our task, responding during the stimulus presentation period did not lead to punishment but did not produce faster rewards either: thus, high-performing animals that became optimally efficient at licking to prompt the reward could have learned to defer licking until the end of the stimulus period. This side-effect of high efficiency could potentially lead to a correlation between latencies and performance that would not involve any variability in deliberation – the animal might have made an early choice of action but deferred licking until the most productive time. To test this possibility, we reasoned that it would result in higher-performing mice (and sessions) having a longer median latency to first lick than impulsive, lower-performing sessions. We thus computed the association between performance and median latency to first lick, on a session-by-session basis. This gave $t = 1.80$, $p = 0.074$ (mixed-effects model with mouse ID as random factor), implying an absence of evidence for a relationship between performance and efficient licking.

Neuronal response analysis

Analyses were conducted in MATLAB and Python. Neurons were first scored visually depending on their responses parsed by trial type (Figures 3, S3).

For each neuronal dataset (S1bf in well-trained and naive animals) we computed PAIRS analyses separately.[42] To do this, we represented each neuron in the dataset by a vector of length 246, constructed by concatenating the cell's average $\Delta F/F_0$ responses to the four trial types with reference to trial time together with the hit and FA trials referred to first lick time. We computed 8 principal components (PCs), which captured over 90% of the variance in the data, and recast each neuron in terms of a response feature vector consisting of the projections of its average response vector onto the 8 PCs. To evaluate response similarity between any two neurons, we took the dot product between their 8-dimensional response feature vectors, which was the cosine of the similarity angle θ between them. For each neuron z, we then obtained the mean of this angle with the neuron's k nearest neighbors, $\theta_z^{(k)}$. The PAIRS measure was computed as the median of $\theta_z^{(k)}$ across all neurons in the dataset, represented as a thick dot in Figures 4A and 5A. To determine whether the response similarity given by the PAIRS measure differs from that expected if occurring by chance, we generated 10000 surrogate neuronal datasets. For every surrogate neuron, the value of each component of the 8-dimensional feature vector was drawn at random from its empirical distribution across real neurons. The plots show results computed for a choice $k = 3$ of the number of nearest neighbors;[42] results were consistent for a wide range of choices of k (3-8).

To check whether the significant clustering present in PAIRS data might be influenced by neurons being grouped according to their session or mouse, we carried out PAIRS analyses separately on data collected from individual experiments, including only sessions where over 25 neurons were imaged. A common PCA basis was used for all experiments within the same category (well-trained or

naive). For all the individual experiments, the experimental PAIRS measure was smaller than the surrogate ones at greater than 95% confidence level.

We based these analyses on fluorescence ($\Delta F/F_0$) time series rather than on activity reconstructed by deconvolution. GCaMP6f does not report linearly on changes in [Ca^{2+}], and in these mice it is often not possible to detect the signal from single action potentials. Moreover, the results of deconvolution methods can be highly sensitive to parameter choice, giving rise to variable conclusions as to neuronal response properties.[124,125] On the other hand, any analysis based on $\Delta F/F_0$ will overestimate neuronal correlations and its estimates on neuronal encoding of task variables are likely to be a smoothed, filtered version of that occurring in reality.[125,126] Our conclusions on clustering of neuronal response properties are not built on absolute estimates of correlations between responses, but on assessments of correlations relative to a random surrogate version of activity. Distortions of response temporal patterning resulting from calcium imaging will tend to smear out differences between neurons, so our results are likely to underestimate the true amount of neuronal heterogeneity.

To estimate how well the activity of a neuron could support classification of trial type (GO versus NOGO, or lick versus no lick), we trained a separate support vector machine (SVM) for each neuron, using as input the $\Delta F/F_0$ time course from all trials, with each labeled according to the trial type of interest. In other words, we used the disparity between the $\Delta F/F_0$ time course over trials of different types to decode whether the trial was either GO versus NOGO or lick versus no lick. To limit bias, if the proportion of trials with licks was more than 75% (a common occurrence in early training), we randomly removed lick trials until the proportion was better balanced than 75%/25%; we only used neurons for which > 100 trials remained after this operation. Training was performed using the sklearn SVM library[116] and classification performance assessed using 5-fold cross validation. A linear kernel was used, and several regularisation parameters tried (.001, 0.01, 0.1, 1) before choosing the one with best cross validated score. The training procedure was repeated for 100 surrogates generated by shuffling trial labels. Neurons were deemed to support classification at a significant level of performance if they performed better than 95 of the surrogates. We repeated this analysis using information theory methods by evaluating the information about trial type conveyed by each neuron's trial-by-trial response, and obtained results qualitatively identical to those based on classifiers.

Current Biology, Volume *31*

Supplemental Information

Sequence Learning Induces Selectivity to Multiple Task Parameters in Mouse Somatosensory Cortex

Michael R. Bale, Malamati Bitzidou, Elena Giusto, Paul Kinghorn, and Miguel Maravall

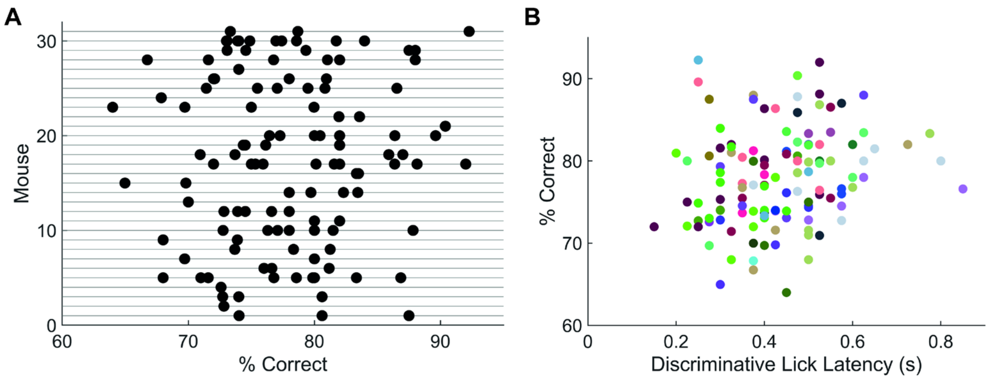

Figure S1. Discrimination of elementary tactile sequences in mice; related to Figure 1. (**A)** Performance (% correct) for all mice and sessions. Performance varied within and across mice. **(B)** Performance plotted against discriminative lick latency across all sessions, as in Figure 1F but with sessions for each mouse displayed in a different colour (n = 31 mice). Variability in performance and latency was present throughout the population of mice rather than being driven by specific animals.

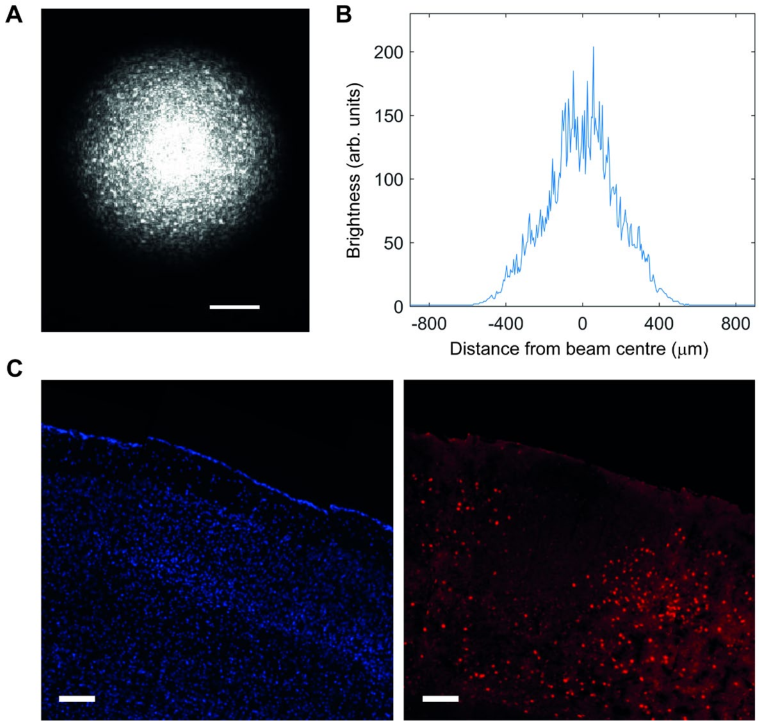

Figure S2. Tracking the participation of different cortical regions with optogenetic activity suppression; related to Figure 2. (A) Focused laser beam spot. Scale bar, 200 μm. **(B)** Measurement of laser beam profile (raw brightness reading, CMOS camera). **(C)** Immunohistochemical characterization of optogenetic suppression in VGAT-ChR2-EYFP mice. Fluorescence in the DAPI (left) and cFos (right) channel for an immunohistochemically processed S1bf slice, after optogenetic stimulation; scale bars, 100 μm. A region with weakened cFos but not DAPI expression can be clearly seen in the middle of the image and measured approximately 500 μm on the side (in the most superficial cortical layers) and 600 μm depth. We took the ratio of fluorescence intensity in the cFos channel and DAPI channel (whose spatially integrated intensity scaled with the density of neurons in an area), and compensated for potential non-specific changes in expression by normalising this ratio in the illuminated hemisphere (shown) to that in the corresponding contralateral region. Within the photoinhibited region, the cFos/DAPI ratio averaged 46% of its value in the contralateral hemisphere.

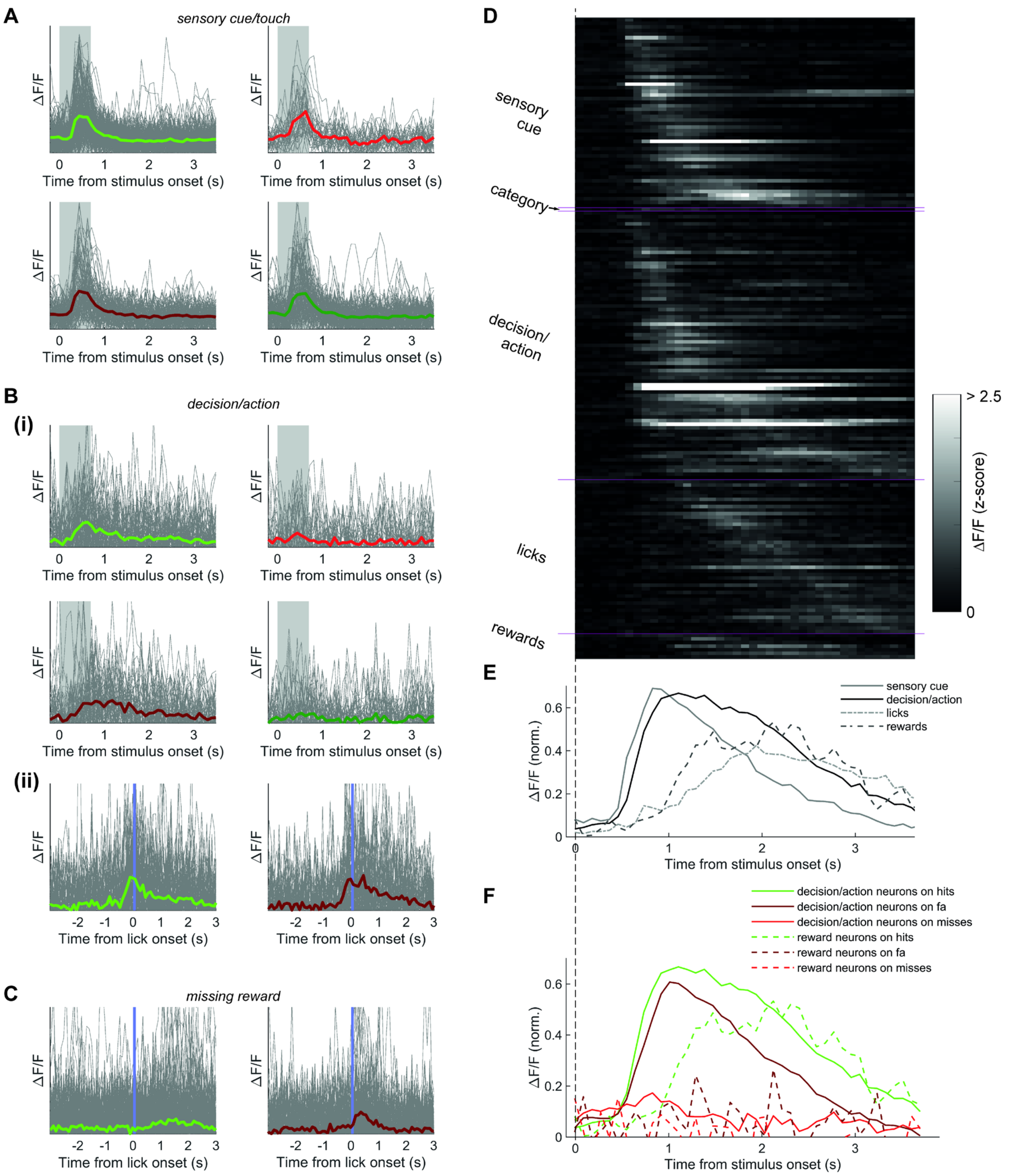

Figure S3. Dynamics and response properties of heterogeneous neurons in well-trained S1bf; related to Figure 3. (A) Trial-by-trial responses of the example 'sensory cue/touch' neuron from Figure 3D. $\Delta F/F_0$ relative to stimulation onset time (shaded region: stimulus presentation period). Thin gray lines, individual trials; thick line, mean. Each panel shows trials of a given type, indicated by the colour of the mean response (bright green, hits; bright red, misses; dark green, correct rejections; dark red, false alarms). Note how, on individual trials, $\Delta F/F_0$ peaks occur predominantly during stimulation, as befits a neuron responding to sensory input. **(B)** Trial-by-trial responses of the 'decision/action' neuron from Figure 3E. Colours as in (A). (i) Responses for all trial types timed relative to stimulus onset. Note how, on individual trials, $\Delta F/F_0$ peaks are spread throughout the trial, as befits a neuron whose responses relate to licks.

(ct'd from previous) (ii) Responses for trials with a lick response (hits and false alarms), timed relative to the onset of the first lick in the 'response period' (i.e. after the end of stimulation) (blue line). Single-trial peaks are seen to cluster around the time of the lick more than around any particular sensory stimulation cue and this gives rise to sharper peaks relative to lick time than stimulus time [compare with plots in (i)]. However, $\Delta F/F_0$ peaks occur throughout the trial, as expected for a neuron whose responses relate to licks. **(C)** Trial-by-trial responses of the 'missing reward' neuron from Figure 3G. Responses for trials with a lick response (hits and false alarms) timed relative to the onset of the first lick in the 'response period'. Colours as in (A). On individual trials, $\Delta F/F_0$ peaks can be found throughout the trial. However, on false alarm trials (right panel), peaks are concentrated just after the time of the first lick that would have been expected to generate a reward: of course, the reward is absent on false alarm trials and this evokes a response. In contrast, on hit trials, most peaks are spread over a much longer period beginning around 1 s after the first lick. This is explained because on any given hit trial, the animal will lick repeatedly until it uses up its water allocation for the trial, at which point the reward for licking will cease. This occurs at a variable time on each hit trial, hence the greater temporal spread of $\Delta F/F_0$ responses compared to the false alarm trials, and the smaller peak in the mean trace. **(D)** Heatmap of response dynamics for all visually classified neurons. Each row plots the mean response on hit trials for one neuron. Neurons are ordered according to their visually scored class (indicated on left), and then according to latency to peak. **(E)** Mean responses to hit trials as in (D), averaged across all neurons in each class. 'Sensory cue' neurons had the earliest response, followed by 'decision/action' neurons which predicted licks relevant to the task, then by 'rewards' neurons which reflected the outcome of task-relevant licks (i.e. reward presence or absence) and by 'lick' neurons which reflected licks regardless of context. **(F)** Comparison between the average responses of 'decision/action' and 'reward' neurons to hit, miss and false alarm trials. 'Decision/action' neurons responded prominently on hit and false alarm trials where the animal licked in response to the stimulus, but not on miss trials where the animal failed to lick to report the GO sequence; in contrast, 'reward' neurons responded prominently on hit trials where the animal licked and was rewarded, but not on miss or false alarm trials.
